# Bionanofactories for Green Synthesis of Silver Nanoparticles: Toward Antimicrobial Applications

**DOI:** 10.3390/ijms222111993

**Published:** 2021-11-05

**Authors:** Ashvi Sanjay Jain, Pranita Subhash Pawar, Aira Sarkar, Vijayabhaskarreddy Junnuthula, Sathish Dyawanapelly

**Affiliations:** 1 Department of Pharmaceutical Sciences & Technology, Institute of Chemical Technology, Nathalal Parekh Marg, Matunga, Mumbai 400019, India; ashvisanjay001@gmail.com (A.S.J.); 18phtps.pawar@ug.ictmumbai.edu.in (P.S.P.); 2 Chemical and Biomolecular Engineering, Johns Hopkins University, Baltimore, MD 21218, USA; asarka11@jhu.edu; 3 Drug Research Program, Faculty of Pharmacy, University of Helsinki, Viikinkaari 5 E, 00790 Helsinki, Finland

**Keywords:** bioreduction, biocapping agent, bionanofactories, biomedical, green synthesis, silver nanoparticles

## Abstract

Among the various types of nanoparticles and their strategy for synthesis, the green synthesis of silver nanoparticles has gained much attention in the biomedical, cellular imaging, cosmetics, drug delivery, food, and agrochemical industries due to their unique physicochemical and biological properties. The green synthesis strategies incorporate the use of plant extracts, living organisms, or biomolecules as bioreducing and biocapping agents, also known as bionanofactories for the synthesis of nanoparticles. The use of green chemistry is ecofriendly, biocompatible, nontoxic, and cost-effective. We shed light on the recent advances in green synthesis and physicochemical properties of green silver nanoparticles by considering the outcomes from recent studies applying SEM, TEM, AFM, UV/Vis spectrophotometry, FTIR, and XRD techniques. Furthermore, we cover the antibacterial, antifungal, and antiparasitic activities of silver nanoparticles.

## 1. Introduction

**Figure 1:**
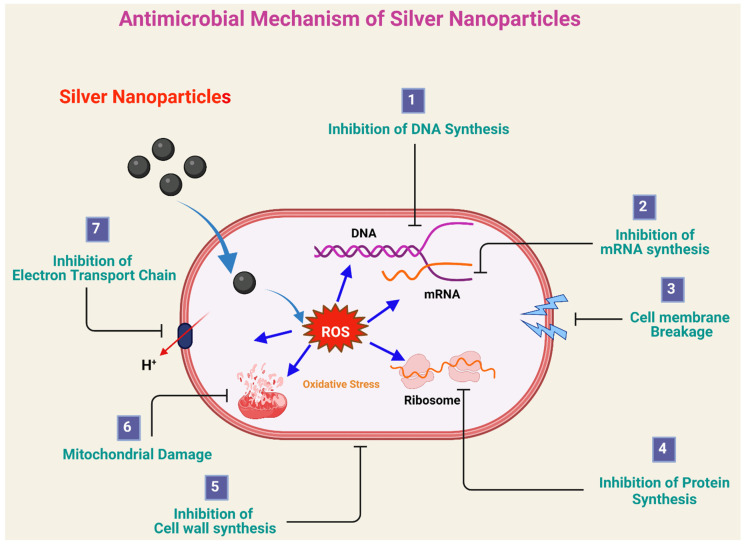
Antimicrobial mechanism of silver nanoparticles: (1) inhibition of DNA synthesis, (2) inhibition of mRNA synthesis, (3) cell membrane destruction and the leakage of the cell constituents, (4) inhibition of protein synthesis, (5) inhibition of cell-wall synthesis, (6) mitochondrial damage, and (7) inhibition of electron transport chain.

Nanotechnology is coming into focus owing to its plethora of applications that can be elucidated as the manipulation of a material using several procedures to create matters with some desired specific properties. It usually involves particles possessing dimensions from 1–100 nm [1]. Their appreciable surface-area-to-volume ratio is the most significant attribute responsible for their extensive use in electronics, nanomedicine, biomaterials, and food [2]. Various physicochemical pathways are employed for the fabrication of nanoparticles (NPs) in the industry. These involve chemical reduction, chemical solution deposition, the sol–gel process, photochemical reduction, and electrochemical reduction. Other methods include laser desorption, sputter deposition, lithographic techniques, layer-by-layer growth, the Langmuir–Blodgett method, the hydrolysis coprecipitation method, the wet chemical method, and catalytic routes [3]. These synthetic approaches compel one to move toward the application of reactive and toxic reducing agents and stabilizing agents, as well as high radiation. However, due to the toxicity associated with them, they adversely affect living organisms, as well as the environment, thus posing various limitations to their use [4]. Hence, to accomplish our needs, over the past decade, efforts have been globally made to reduce the generation of hazardous waste. Green synthesis can be accomplished by adopting the 12 fundamental postulates of “green chemistry” proposed by Anastas and Warner and integrating them with modern developments. This can reduce the use of harmful chemicals and elevate the efficiency of the process [3,5]. Hence, green synthesis is a possible alternative to harsh physical and chemical operations. The use of nontoxic solvents [6,7,8,9] and sustainable materials represents crucial components that need attention in this ecofriendly approach [10]. Several factors influence the choice of the green approach over conventional methods. The term “green” is not the color, but the concept of synthesizing nanoparticles from metal salts by exploiting the reducing property of biologically active compounds. These biologically active compounds may be obtained from microorganisms (both live and dead), herbal extracts (from leaf, root, whole body, flower, fruit, bark, latex, etc.), and animal extracts [11]. Nanoparticles derived from biological materials are known as biogenic nanoparticles, and the involved synthesis process is known as the green synthesis of nanoparticles [2]. This concept of NP synthesis was earliest proposed by Raveendran et al. by employing β-d-glucose as a reducing agent and starch as the capping agent in the preparation of silver nanoparticles (AgNPs) [5]. The specific antimicrobial mechanisms of AgNPs still remain unknown. According to research investigations, the expected antimicrobial activity of silver nanoparticles are proposed in Figure 1. AgNPs releases Ag^+^ ions, which can accumulate on the cell wall and cell membranes of microorganisms and further enter into cytoplasm. Inside the cell, Ag^+^ ions generate reactive oxygen species (ROS), which are the key agent for antimicrobial activity, involving (1) inhibition of DNA synthesis, (2) inhibition of mRNA synthesis, (3) cell membrane destruction and the leakage of the cell constituents, (4) inhibition of protein synthesis, (5) inhibition of cell-wall synthesis (6) mitochondrial damage, and (7) inhibition of the electron transport chain. These effects eventually lead to cell death. In addition to being able to release silver ions, AgNPs can themselves kill bacteria.

The antimicrobial properties of AgNPs are well reported in the literature; however, the safety of these particles and surface cytotoxicity issues in living cells are of a concern for their use in biomedical applications. Recently, Barbalinardo at al. studied the surface cytotoxicity of AgNPs after surface modulation using an oligo (ethylene glycol)-based ligand (11-mercaptoundecyl)hexa(ethylene glycol(EG6OH)) and concluded that the cytotoxicity of nanoparticles was reduced with an increase in ligand density; they further stated that rational design and engineering could potentially minimize the side-effects of the AgNPs [12]. Furthermore, EG6OH-coated AgNPs were not internalized and did not show any cytotoxicity in mouse embryonic fibroblast (NIH-3T3) cells [13]. These studies revealed that the relationship among surface coatings, ligand density, and protein corona formation characteristics are useful in modulating surface properties for better use of AgNPs.

The green chemistry perspective involves the three main steps generally involved in the preparation of nanoparticles. These include the selection of a solvent medium, focusing on the selection of a “green” alternative for the reducing agent and a harmless substance to stabilize the NPs, as a vast number of conventional methods depend on organic solvents, majorly contributing to the hydrophobic properties of the capping materials involved in the process. Another concern is the selection of a reducing agent. Most processes reported until now mostly employ hydrazine, dimethyl formamide (DMF), and sodium borohydride (NaBH_4_) as reducing agents, although they are highly reactive and pose various environmental issues [5]. Hence, the chemical reduction methods can be replaced by biogenic reduction, which is a “bottom-up” technique, wherein the extract of a natural product that possesses innate properties of stabilizing, growth-terminating and capping of NPs replaces the harmful and toxic reducing agents [11]. Furthermore, this method seems to be more atom-efficient as the particles are built atom-by-atom in the process and do not require the use of protection/deprotection processes used in a traditional organic approach [10]. The final challenge to be solved is to select an appropriate capping material that can be employed to make the nanoparticle surface unreactive (passivation). The choice of capping agent is influenced by various challenges that exist in the process of synthesis and vary with numerous factors. However, those substances utilized as reducing and stabilizing agents include proteins, enzymes, sugars, and certain phytochemicals such as flavonoids, terpenoids, and cofactors in these green synthesis methods [5]. This helps to produce NPs which are environmentally friendly, low-cost, and nonpolluting especially for healthcare and biology applications that demand high-grade purity [11]. Hence, green synthesis is advantageous as it is economical, environmentally friendly, and uncomplicated for large-scale synthesis as the plants and their extracts employed represent promising solutions due to their availability, suitability for mass production, and environmentally benign nature. Moreover, this approach does not demand employing various industrial processes for maintaining high temperature, pressure, or energy requirements [14]. Green NPs are synthesized in a one-step procedure that is advantageous for controlling and manipulating the crystal growth, stabilization, and particle size and shape. This single-step reduction technique requires a lower amount of energy for synthesis as the processes are operated at near-ambient temperature, pressure, and pH, which again follows the principles of green chemistry [3]. This also helps to prevent particle toxicity and reactivity toward our health and the environment, as a lack of predictability and composition ambiguity of the nanoparticles are not experienced. Moreover, expensive metal salts such as gold and silver can be recycled from the waste generated by applying these green fabrication strategies, thereby controlling the issue of limited reserves and high prices of these metals. Thus, green chemistry is aimed at thwarting waste, using sustainable materials, and employing techniques that lower the risk to living beings, which is accomplished via the green synthesis of nanoparticles [10].

## 2. Strategies for Green Synthesis of Silver Nanoparticles

**Figure 2:**
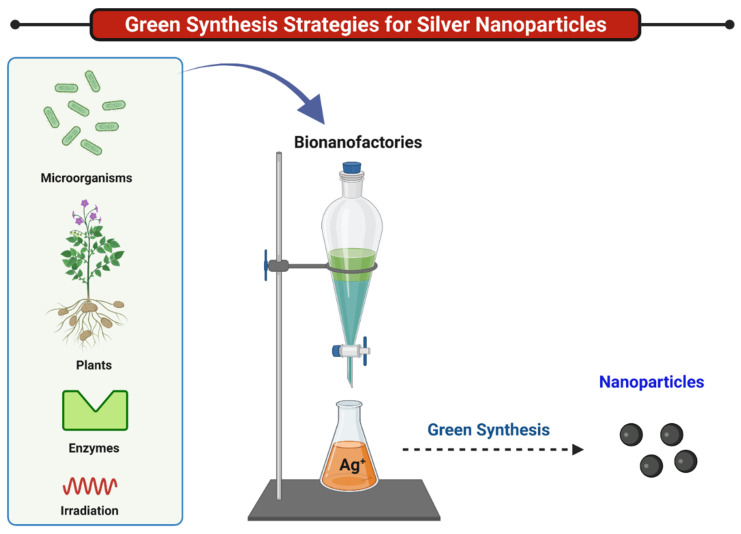
Green synthesis of nanoparticles of silver nanoparticles.

Numerous classes of microorganisms and plants have been employed to successfully accomplish the green synthesis of NPs. These methods of synthesis have also garnered importance as an alternative strategy for the development of gold, silver, zinc, titanium, and palladium NPs. Several reviews have been reported on the methods for biosynthesis of NPs [15] that majorly deal with plants [16,17] microbes [18], marine organisms [19], and phototrophic eukaryotes. The potential microbial bionanofactories that can be utilized to synthesize NPs intra- or extracellularly include bacteria, algae, yeast, and fungi [20]. Different parts of the plant including leaves, stem, bark, root, fruit, and flower can be selected for this new ecofriendly approach of synthesis. This vast range of phytochemicals includes *Aloe vera* plant extract [20], *Mangifera indica* fruit extract [21], *Murraya koenigii* leaf [22], and others carbohydrates [23]. Glucose was also utilized to synthesize AgNPs along with the appearance of stabilizing agents such as soluble starch [5,24,25], sucrose, and maltose [26]. One of the very well-known plants is *Eucalyptus* [27], whereas *Bacillus methylotrophicus* [28] has also attracted interest in green synthesis. Ubiquitous plant materials such as *Coffee arabica* seeds [29] and *Azadirachta indica* [30] have also played a significant role in extensively utilizing plant materials in this process of green synthesis. Even peanut shells have been trialed for green silver NP synthesis [31]. Spices such as pepper leaf extract were reported in [32] as an example of the green synthesis of nanoparticles. Other synthesis methods utilizing various parts of plants are summarized throughout this review. The higher degree of safety and stability, as well as biocompatibility, offered by these green NPs is due to their surface being capped with nontoxic biomolecules. Moulton et al. noted that polyphenols as capping agents can potentially impart superior antioxidant effects to the synthesized AgNPs [33]. Furthermore, studies have found that the metabolites present in plant extracts, such as proteins [34] and chlorophyll [35], act as the capping agents for synthesized AgNPs. Figure 2 represents the strategies of green synthesis and the use of bionanofactories in the synthesis of silver nanoparticles, and their physicochemical properties are briefly outlined in Table 1.

### 2.1. Phytosynthesis

Plants have always been exploited by humans since the Stone Age for their metabolites, which have proven to be a pillar of human survival. Similarly, there have been numerous experiments for this emerging method of NP synthesis that can enhance the potential applications of the plants and their extracts in this field. Many studies have shown that phytoconstituents such as flavonoids, terpenoids, pectin, sugars, ascorbic acid, and carotenoids present in powders or extracts of roots, shoots, bark, leaves, peel, flowers, and fruits can function as reducing and capping agents to develop NPs [66].

For in vitro green synthesis, the required chemical metabolites are first extracted from plant organs and then suitably incubated with NP precursors to produce NPs. The obtained NPs are subject to centrifugation and washing, allowing them to be collected. Furthermore, the NPs are characterized by employing various methods, and studies for analyzing the release of Ag^+^ from the AgNPs are also conducted. In one such study mentioned [67], it was observed that, after entering the aquatic environment, AgNPs would release silver ions, which would decrease the stability of the AgNPs. Furthermore, Lee et al. suggested that the release of Ag ions follows first-order kinetics [68]. There are several factors affecting the release rates of Ag ions that must be considered while evaluating these synthesized NPs; they mainly include particle size, environmental factors, e.g., pH, temperature, and dissolved oxygen [69,70], and capping agents [71]. However, it has been observed that silver ions exhibit different physiochemical properties and biological toxicity from the synthesized AgNPs; hence, detailed studies are necessary before these AgNPs are put into real-life application [69]. In another interesting study, AgNP synthesis was reported by Forough et al. utilizing two plants, wherein an aqueous extract of soap-root (*Acanthe phylum bracteatum*) and an aqueous manna extract of *Hedysarum* were employed as the stabilizing agent and reducing agent, respectively. Manna has been widely used in Asia as it possesses laxative properties [72].

#### 2.1.1. Extracts of Roots

**Figure 3:**
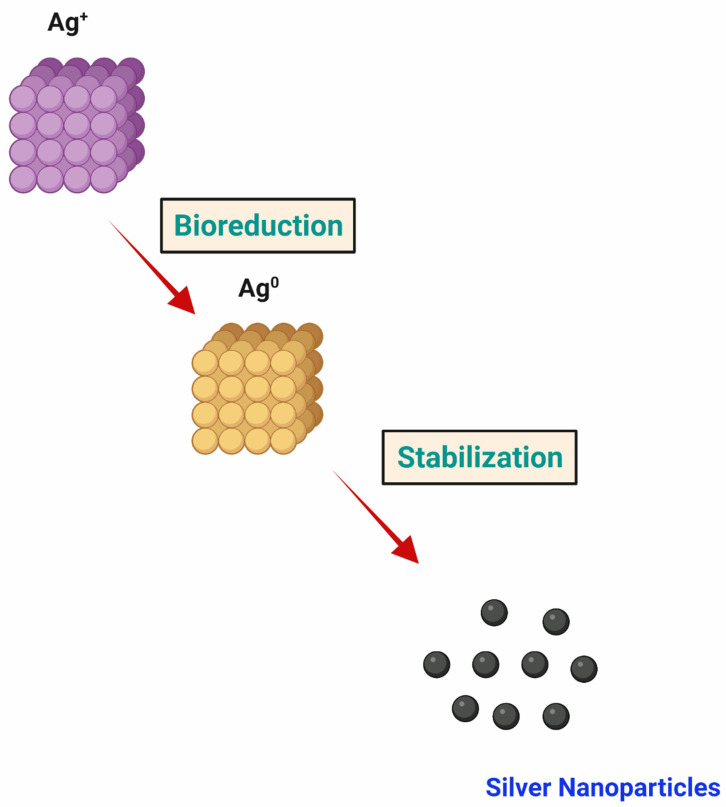
Green synthesis of silver nanoparticles from bioreduction of silver ions. The first step involves the bioreduction of positive Ag^+^ into the zero-valent Ag^0^ metal, while the last step involves the stabilization of metal NPs.

The synthesis of metallic nanoparticles using *Medicago sativa* [73] is perhaps one of the earliest records on the generation of AgNPs utilizing a plant part as a source. Alfalfa roots absorb the reduced silver (Ag^+^ to Ag^0^) from agar medium and transmit it to the shoots in the identical oxidation state (Ag^0^). Then, Ag atoms in the shoots organize themselves by joining together and forming larger arrangements to produce NPs. TEM/STEM analysis displayed the aggregation of Ag atoms in the interior of the plant tissue, whereby they underwent nucleation and NP formation. An aqueous root extract of *Parthenium hysterophorus* has been employed to reduce silver ions and synthesize stable green NPs, which further showed larvicidal activity toward *Culex quinquefasciatus* in mosquito control [74]. Figure 3 shows the bioreduction of silver ions into silver nanoparticles.

#### 2.1.2. Extracts of Seeds

To illustrate AgNP synthesis using plant seeds, Bar et al. described the fabrication of green silver NPs by utilizing the seed extract from *Jatropha curcas* [75], wherein it was noted that the major phytoconstituents including curcain (an enzyme), curcacycline A (a cyclic octapeptide), and curcacycline B (a cyclic nonapeptide) could be employed as reducing and capping agents. The resultant NPs were further evaluated and characterized by HR-TEM, XRD, and UV–Vis spectroscopy. The analytical results showed two broad distributions of AgNPs, among which those having a diameter from 20 to 40 nm possessed a spherical shape, whereas the other particles were found to be larger and uneven in shape. It was further observed that the cavity of the cyclic peptides (curcacycline A and curcacycline B) stabilized the smaller NPs, while the sizeable ones were stabilized by the enzyme curcain. This interpretation was based on the demonstration that the peptides of proteins or carbonyl groups of amino-acid residues have strong metal-binding affinity [76]. Hence, the protein can protect the NPs by preventing their agglomeration, thus working as an encapsulating agent. It was believed that the cyclic proteins, curcacycline A or curcacycline B, first entrapped the Ag ions in their core structure. The subsequent reduction and stabilization of AgNPs happened in situ by the amide groups of the host peptide under suitable process conditions. Since the radius of most AgNPs obtained was comparable to the cavity of cyclic peptides, it was considered that the cyclic peptide cavity stabilized the smaller AgNPs, whereas the irregularly sized AgNPs were stabilized by the enzyme curcain, owing to its large, folded protein structure. Studies also concluded that the AgNPs synthesized by curcain latex were stable even after 1 month [77].

Vidhu et al. demonstrated the application of *Macrotyloma uniflorum* for AgNP synthesis. The plant is commonly known as horse gram, which is an herbaceous type of plant having a numerous pharmaceutical properties [77]. Several plant parts such as seeds and leaves are used for the treatment of asthma, heart conditions, bronchitis, urinary discharges, leukoderma, etc. Seeds of horse gram are a good source of molybdenum, iron, phenolic compounds, and antioxidants. The phytochemical analysis of the constituent seed indicated the presence of different phenolic acids such as 3,4-dihydroxy benzoic acid, *p*-hydroxy benzoic acid, vanillic acid, sinapic acid, syringic acid, caffeic acid, ferulic acid, and *p*-coumaric acid. The carbonyl and hydroxyl groups present in phenolic compounds were found to be capable of binding to metals [77]. Such compounds help in the inactivation of ions via the process of chelation. One of the constituents of the seed, i.e., caffeic acid, is thought to possess great antioxidant activity as it possesses an added conjugation in its propanoic side-chain that favors the delocalization of electrons via resonance. Here, it is synthesized from 4-hydroxy cinnamic acid in plants and is converted to ferulic acid after the release of a hydrogen. It is believed that this active hydrogen mediates the reduction of silver ions that form the resultant AgNPs. Hence, it is believed that the presence of proteins and phenolic compounds may act as the strategic factors for the synthesis of AgNPs. Hence, it is evident that this NP synthesis method utilizing the biochemical approach, by employing several plant extracts to play a key role as reducing and capping agents, is potentially a promising method for future developments on similar lines.

#### 2.1.3. Extracts of Fruits

As per the literature, fruits have played a major role in ecofriendly AgNP synthesis. Amin et al. employed fruit extract from the *Solanum xanthocarpum* plant for the reduction and capping of AgNPs. It is a thorny plant known as Indian nightshade or yellow-berried nightshade, which grows in various terrains of the Indo-Pakistan subcontinent. These fruits are a rich source of apigenin glycosides, quercitrin, and flavonoids, and their extract displays antimicrobial, antioxidant, and anthelmintic properties [78]. This study concluded that pH, temperature, and the molar ratio of AgNO_3_ to *S. xanthocarpum* extract (SXE) influence the reduction of Ag^+^ and size of AgNPs. These fabricated particles exhibited urease-inhibitory and anti-*H. pylori* activities; accordingly, the study hinted at the potential antibacterial and urease-inhibitory activities of the AgNPs. This synthesis route for the AgNPs utilized SXE extract with AgNO_3_ at 45 °C for 25 min, resulting in a band centered at 406 nm with surface plasmon resonance (SPR). These synthesized particles were observed to be spherical and monodispersed in nature with a size of around 10 nm. These NPs displayed appreciable effectiveness against the antibiotic-susceptible and antibiotic-resistant strains of *H. pylori.*

Indian gooseberry (*Emblica officinalis*) fruit extract was employed as a reducing agent to fabricate AgNPs [76]. In the study, treatment of aqueous chloroauric acid solution and silver sulfate with *Emblica officinalis* fruit extract, resulting in the reduction of Ag^+^ ions into highly stable NPs. TEM analysis of the AgNPs reported here demonstrated that they were approximately 10–20 nm in size.

Li et al. reported the synthesis of AgNPs using *Capsicum annuum* extract. Studies suggested that the relationship between recognition–reduction–limited nucleation and growth is essential to elucidate the mechanism of formation of AgNPs. The first step was the recognition step wherein the proteins present in the *Capsicum annuum* extract interacted with the Ag ions through electrostatic interactions [79]. Then, Ag ions were reduced by proteins in the extract, which resulted in the generation of silver nuclei, as well as caused variations in the secondary structures of proteins. Moreover, the reduction of silver ions and their further accretion on these nuclei resulted in their subsequent growth. Larger AgNPs were formed with an increase in time. The polycrystalline phase turned into single crystalline phase via Ostwald ripening due to the increase in aging time, resulting in large-sized AgNPs.

In another study, a plant from the Bromeliaceae family, *Ananas comosus L.* (pineapple) [80], which has several beneficial properties including antioxidant activity, was used to produce AgNPs using the juice of the pulpy fruit. Phenolic bioactive constituents, present in vegetables and fruits, have been found to be majorly responsible for health benefits [81]. Ferulic acid in pineapples is believed to act as a reducing agent, which is oxidized by AgNO_3_, further leading to the formation of the AgNPs. Another phenol known to be present in pineapple extract is chlorogenic acid. Such antioxidants present in pineapple juice act synergistically as reducing agents and stabilizing agents for silver metal ions.

#### 2.1.4. Extracts of Leaves

Medicinal herbs such as *Hibiscus rosa sinensis* are effectively utilized in the treatment of hypertension, pyrexia, liver disorder etc. Philip et al. successfully used the above for AgNPs synthesis, wherein a quick change of the solution color to golden yellow indicated the formation of AgNPs. Its leaf extract contains antioxidant compounds and certain organic acids (essentially malic acid), proteins, flavonoids, anthocyanins, and vitamin C.

Interestingly, in another examination, Singh et al. [82] demonstrated the reduction of Ag ions present in a silver nitrate solution with the application of an aqueous extract of *Argemone mexicana* leaf. The color of the aqueous solution of the Ag ions changed from watery to yellowish brown due to the reduction of silver ions, indicating nanoparticle formation after the mixing of the *Argemone* leaf in the complex [74]. Studies suggested that the biosynthesized NPs are extremely toxic against various pathogenic fungi and bacteria at a concentration of 30 ppm for their growth control. The results from SEM and XRD studies displayed that the particle size range was 25–50 nm, and they were cubic in structure. The fact that the bioreduction of Ag^+^ ions to AgNPs was due to the capping action of the plant extract was further confirmed by FTIR analysis [83].

The literature suggests that the plant extract from *Ocimum sanctum* (Tulsi) can be a good source of stabilizers, as well as bioreducing agents. Studies have shown that the glycosides, alkaloids, saponins, and tannins contained in the extract can be used to treat diarrhea, headaches, worms, and cough. Jain et al. employed green synthesis strategies to develop stable AgNPs using a leaf extract of quercetin and tulsi. TEM micrographs of AgNPs indicated the spherical and unform size of NPs with quercetin (11.35 nm) and tulsi (14.6 nm) as reducing and capping agents. In the case of quercetin, the size of nanoparticles was increased from 11.35 nm to 18 nm upon increasing the pH to 10 [84].

Mallikarjuna et al. developed nanoparticles 3–20 nm in size, which were characterized using TEM, XRD, UV/Vis spectroscopy, and FTIR techniques [85]. Moreover, these reduced AgNPs were covered with proteins and metabolites, e.g., terpenoids, with the functional groups of carboxylic acids, amines, ketones, alcohols, and aldehydes. The FTIR studies showed that the carbonyl groups from the amino-acid residues and proteins possess significant potential to bind metal, suggesting that the proteins (perhaps from the metal NPs i.e., capping of AgNPs) could put a stop to the aggregation that stabilizes the medium. This experiment hinted at the ability of the biological molecules to display binary actions in aiding the formation and stabilization of AgNPs in the aqueous medium.

**Figure 4:**
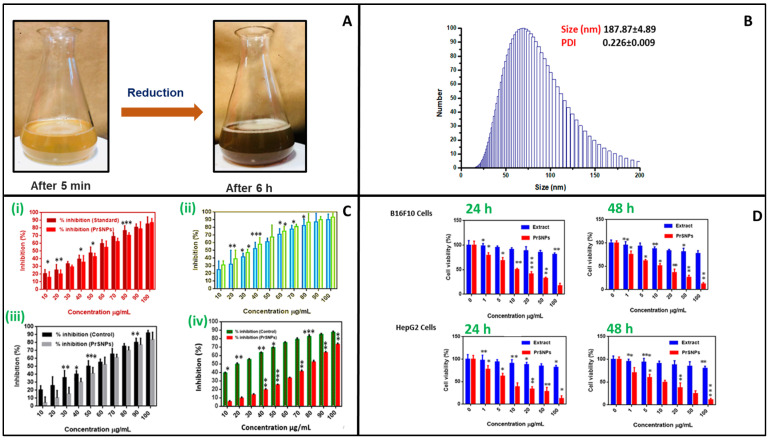
Green synthesis of AgNPs using leaf extract of *P. hysterophorus*. (**A**) The reduction of AgNO_3_ by *P. hysterophorus* extract indicated a color change after 5 min and 6 h. (**B**) Particle size distribution. (**C**) Anti-inflammatory activity of AgNPs according to (i) DPPH assay, (ii) H_2_O_2_ assay, (iii) NO free radical-scavenging assay, and (iv) nitric oxide radical-scavenging assay. (**D**) In vitro cytotoxicity test on B16F10 and HepG2 cell lines after 24 h and 48 h treatment; * *p* < 0.05, ** *p* < 0.01 and *** *p* < 0.001, unpaired Student’s *t*-test. CC-BY License [86].

In one study, a leaf extract of *Parthenium hysterophorus* was employed for the optimized green synthesis of AgNPs with an average particle size of 187.87 ± 4.89 nm and zeta potential of −34 ± 3.12 mV (shown Figure 4). A significant anti-inflammatory activity of NPs was observed. In addition, the in vitro cytotoxicity of AgNPs displayed potential anticancer activity after treatment of B16F10 and HepG2 cell lines at 24 h and 48 h. This leaf extract of *Parthenium hysterophorus*-based AgNPs could be a promising *Candida*te as an antimicrobial, antioxidant, anti-inflammatory, and antitumor agent for treatment [86].

Jha et al. reported another strategy involving the extract of *Cycas revoluta* (family Cycadaceae) [87], the source of sago. It is considered to be a rich source of fatty acids such as palmitic, stearic, oleic, and behenic acids, as well as flavonoids. Flavonoids are basically phenolic compounds which are found in almost all vascular plants. Amentiflavone and hinokiflavone are present in *Cycas* leaves as characteristic biflavonyls, which again act as reducing agents. AgNO_3_ solution was treated with this leaf broth for 4 h, and their images were recorded by TEM, displaying discrete spherical nanoparticles possessing a diameter of around 2–6 nm. To ascertain the crystal structure of AgNPs, XRD was applied, with the lattice parameter showing good agreement with previous publications. There was an immediate change in color wherein the extract turned yellowish brown after adding the *Cycas* ethanol extract to the AgNO_3_ solution. Here, the generation of AgNPs occurred as a result of reduction, after which UV/Vis spectroscopy at 449 nm was conducted on the resultant AgNPs [87].

Another study designed a cost-effective, simple, and green synthesis method of AgNPs using an extract of mulberry leaves as a reducing and stabilizing agent. The generated NPs had a mean size of 20 nm and possessed a face-centered cubic (FCC) structure. It is quite evident that plants are “biofactories”, as the rate of synthesis of NPs with the application of plant products/extracts is faster than that when using microorganisms, while the resultant NPs are also more stable [88].

Other recent green methods to obtain AgNPs include those given by [89] employing *Olea europaea* leaf extract. These were synthesized by utilizing hot-water olive leaf extracts (OLE) that function as a reducing and a stabilizing agent; they were further tested for activity against drug-resistant bacteria. The spherical AgNPs synthesized possessed an average size of 20–25 nm. The AgNPs at 0.03–0.07 mg/mL concentration appreciably inhibited bacterial growth of multidrug resistant *Pseudomonas aeruginosa (P. aeruginosa)*, *Staphylococcus aureus (S. aureus)*, and *Escherichia coli (E. coli)*. This study also highlighted that the aqueous olive leaf extract exhibited no notable effect at the concentrations utilized for the preparation of these NPs. Elavazhagan et al. [90] developed AuNPs and AgNPs using *Memecylon edule* leaf extracts, which is a shrub also known as iron wood tree. According to various studies, saponins are the most favorable phytoconstituents for NP synthesis. Some studies have also demonstrated that the application of plant extracts for AgNP synthesis is faster than fungi- or bacteria-mediated synthesis [91]. For instance, Shankar et al. demonstrated the synthesis of NPs utilizing *Pelargonium* leaf in 9 h, while synthesis took about 24 to 124 h for the previously mentioned reactions [35].

The literature suggests that one of the major advantages of phytosynthesis is the easy handling of plant compounds or extracts and ready accessibility. Furthermore, the plants possess several active agents that facilitate Ag ion reduction, augmenting the synthetic procedure. It has also drawn attention since this route is capable of providing an economical ecofriendly protocol that occurs in a single step via a nonpathogenic synthesis route [92].

It has also been reported that centrifugation [83] can be used to obtain the AgNPs in pellet or powder form. In the case of the formulation of AgNPs suspensions, the product can be obtained in powder form via oven-drying [93]. Today, most parts of the plant such as roots, latex, stem, leaves, flowers, and seeds are used for NP synthesis. The most important factor to be kept in mind is the presence of bioactive agents in these parts responsible for the reduction and stabilization of nanoparticles. The medicinal plants used for AgNP creation are useful for control over the size and shape of the particles; in addition, such plants also offer their antimicrobial properties to the synthesized NPs. The utilization of plant extracts for the green synthesis of NPs could also be beneficial compared to other green synthesis processes, as these do not demand the intricate process of nurturing cell cultures as required in cases of microbial synthesis. A group of researchers also developed AgNPs using various plant leaf extracts such as *Aloe vera* [85], *Cinnamomum camphora* [85], *Camellia sinensis* [94], *Diopyros kaki* leaf, *Magnolia kobus* [95], *Geranium* leaf [35], *Acalypha indica* leaf [96], *Coriandrum sativum* [97], *Sorbus aucuparia* leaf [98], *Gliricidia sepium*, and rose leaf [99].

### 2.2. Microbial Synthesis of Silver NPs

Various types of microorganisms have been explored as biofactories for the synthesis of green NPs, and these strategies are extensively discussed in the literature [18,100]. Ahluwalia et al. demonstrated the utility of the fungus *Trichoderma harzianum* for AgNP synthesis, in addition to its extensive use as an agricultural fungicide [101]. The efficiency of the method was proven by the formation of NPs that are stable beyond 3 months of manufacturing. In addition, various types of broths such as lysogeny broth, peptone broth, nutrient broth, yeast extract, yeast mold broth, and tryptic soy broth have also been investigated for synthesis [102]. The formation of NPs is majorly impacted by two critical parameters, broth pH and light condition [102]. There are two mechanisms involved in the microbial synthesis of AgNPs [103]: intracellular synthesis and extracellular synthesis. In the intracellular method, the enzymes and the other biomolecules present inside the microbial cells are accountable for Ag ion reduction to NPs [2], and nucleation of the synthesized AgNPs is caused by the accumulation of Ag inside the cell, wherein the process continues with the growth of microbes. Once the optimum growth of cells is achieved, the live cells are harvested. Furthermore, special treatment procedures are employed for release of the synthesized NPs from the harvested cells [103]. In a study, Otari et al. demonstrated the intracellular synthesis of AgNPs using *Rhodococcus* spp. When tested against pathogenic microorganisms such as *Pseudomonas arugenosa*, *Stapylococcus aureus*, *Klebsiella pneumoniae*, *Enterococcus faecalis*, and *Escherichia coli*, fabricated AgNPs were found to exhibit great bacteriostatic and bactericidal activity. In contrast to the conventional physical and chemical methods of synthesis of AgNPs, this approach offered a cheaper and greener route of synthesis with scope for bioremediation [104]. In the extracellular method, the synthesis is done using extracellular secretions of the bacterial cells, and this method offers added advantages over the intracellular method, such as ease of separation along with the absence of downstream processing protocols [2]. In another study, Singh et al. (2014) revealed the extracellular biosynthesis of AgNPs using *Penicillium* spp., isolated from *Curcuma longa* (turmeric) leaves. The synthesized silver NPs showed appreciable activity against multidrug-resistant bacteria such as *Staphylococcus aureus* and *Escherichia coli* [105]. As discussed earlier, biosynthetic methods mediated by microbes can be categorized into extracellular and intracellular synthesis according to the location of NP production. Of these methods, the extracellular synthesis of NPs is still under study to comprehend the mechanisms employed for synthesis and a rapid scale-up. The intracellular mechanism for the green synthesis of metallic NPs has also been investigated [15] by using various types of plant and microbial species [16,17]. This microbial synthesis includes a range of reactions which involve trapping, bioreduction, and capping. The enzymes present in the cell wall of microbial species reduce the metal ions [18]. The intracellular synthesis of nanoparticles has several limitations such as low production and difficult purification [17].

However, there are several drawbacks of microbe-mediated NP synthesis. These include the expenses associated with upstream and downstream processing, making it an expensive resource-intensive synthetic route. Additionally, this method seems less feasible for industrial application because microbial cells demand an extremely specific environment for optimal growth. It was also found that, although microorganisms possess resistance mechanism against Ag ions, which is beneficial for the effective production of AgNPs, they show varying degrees of such resistance depending on the organism. Such resistance aids in the synthesis of AgNPs at a high concentration without killing the microbial cells. Generally, with increasing concentration, the rate of cell death increases [103].

#### 2.2.1. Bacteria-Mediated Green Synthesis of Silver NPs

Among the various classes of microbes [106], the use of bacteria is gaining importance and is prevalent because of its easy and extensively studied genetic modification protocols, simple handling, and rising accomplishments [107]. Bacteria are regarded as promising *Candida*tes for this ecofriendly route of synthesis, which is attributed to their intrinsic potential to reduce heavy metals. Several factors such as organic functional groups present in the bacterial cell wall work synergistically to carry out the reduction [108]. In one such study [109], *Enterococcus* species isolated from fermented foods and further extracts of various strains (*n* = 6) were employed for the generation of nanoparticles. The prepared NPs displayed antimicrobial activity against multidrug-resistant species including *E. coli*, *K. pneumoniae*, and *P. vulgaris*. In addition, these NPs showed synergistic antimicrobial activity with ampicillin, ciprofloxacin, and cefuroxime. Thereafter, these NPs were used as nanopreservatives in white emulsion paint [109].

In another investigation, Sunkar and Nachiyar et al. synthesized AgNPs using endophytic bacterium *Bacillus cereus* isolated from the plant *Garcinia xanthochymus*, which is also known as false mangosteen or Himalayan *Garcinia*. The obtained nanoparticles were spherical AgNPs with their size in the range of 20–40 nm. The studies also demonstrated that the synthesized NPs possessed augmented activity against pathogenic bacterial species such as *Klebsiella pneumoniae*, *Salmonella typhi*, *Escherichia coli*, *Staphylococcus aureus*, and *Pseudomonas aeruginosa* [110].

Various other demonstrations have been reported, wherein the bacteria were employed to synthesize AgNPs; for example, [106] utilized *Pseudomonas stutzeri* AG259 obtained from a silver mine for the fabrication of silver NPs with a well-defined size and sharp morphology, including shapes such as equilateral triangles and hexagons. The study also revealed that the characteristics and morphology of nanoparticles can be mediated by several factors including cultivation conditions such as the time of incubation, composition and pH of growth media, and exposure to light [111].

**Figure 5:**
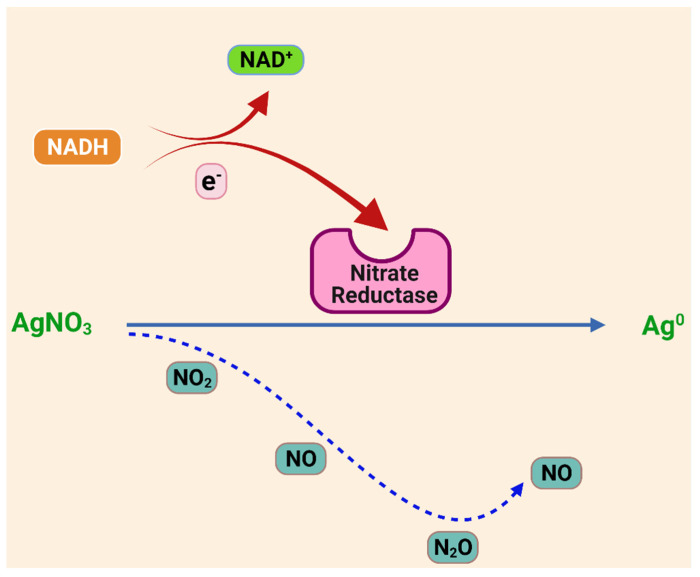
Nitrate reductase-mediated biogenic synthesis of AgNPs.

Karthik et al. demonstrated the extracellular synthesis of AgNPs using bacterial species *Streptomyces* sp. LK3. The hypothesized mechanism involves a reduction of nitrate to nitrite by nitrate reductase, and this mechanism is widely accepted [112]. Some studies have also suggested that the NADH-dependent nitrate reductase-mediated reduction is the key factor in the green synthesis of AgNPs (shown in Figure 5). In the process of reduction, the electron is transferred to the Ag^+^, leading to its reduction to metallic Ag [113]. The green synthesis of AgNPs was investigated using nitrate reductase (NR) from *Fusarium oxysporum* [113,114]. In another study, it was also observed that the chemical functionalities of the bacterial cell wall reduced silver ions to metallic silver in the absence of NR enzyme [103].

The synthesis of AgNPs using *Plectonema boryanum* UTEX 485, a filamentous cyanobacterium, revealed the participation of proteins during synthesis [115]. The culture supernatants of *Escherichia coli*, *Enterobacter cloacae*, and *Klebsiella pneumoniae* have also resulted in speedy formation of AgNPs [116]. Shahverdi et al. studied the biosynthesis of AgNPs using culture supernatants of different strains of enterobacteria including *Bacillus cereus*, *Bacillus subtilis*, *Escherichia coli*, *Enterobacter cloacae*, *Klebsiella pneumoniae*, *Lactobacillus acidophilus*, *Staphylococcus aureus*, *Pseudomonas aeruginosa*, *Candida albicans*, *Aspergillus Niger*, and *Enterobacter cloacae* [109,116]. In this approach, culture flasks of enterobacteria were incubated at 35 °C for about 24 h, and this rapid process led to the formation of AgNPs in just 5 min of contact with the culture supernatants. Extensive scientific research has been conducted to study the extracellular green synthesis of AgNPs using *P. aeruginosa* [117] and *E. coli* [118,119]. Studies suggest that silver-resistant bacterial cell walls can accumulate a maximum amount of silver at 25% of their dry weight biomass [107,120]. The flexibility and cost of bacteria-mediated biosynthesis are dependent on the selection. This was also reported as a suitable method for large-scale production [121]. However, a major setback in the biosynthesis of NPs using bacteria is the time-consuming synthesis procedure and the limited availability of sizes and shapes compared to other methods of synthesis. Due to this reason, fungi-based nanofactories or plant-based materials were further examined in [122].

#### 2.2.2. Fungi-Mediated Green Synthesis of Silver NPs

Considering the limitations of the bacteria-mediated synthesis [106], a plethora of different proteins, the cell mass, the enzymes, or the extracellular components from fungi, such as *Aspergillus flavus*, *Fusarium oxysporum*, *Penicillium brevicompactum*, and *Aspergillus clavatus* [123,124], have been investigated to reduce silver ions in order to synthesize AgNPs. Around the beginning of the 20th century, the first fungi-mediated metal nanoparticle generation was reported as *Verticillium*-mediated AgNP synthesis with an average diameter of about 25 ± 12 nm [125,126], as reviewed in [91]. The suggested pathway for the synthesis of NPs includes a few steps. The first step involves the electrostatic interaction between COO^−^ groups present in the enzymes of the mycelial cell wall and Ag^+^ ions, leading to the absorption of positively charged silver ions on the fungal cell surface. The second step is the reduction of Ag^+^ ions by the enzymes present in the cell wall, followed by the generation of silver nuclei, which eventually form the AgNPs [127]. In another example [106], the superiority of filamentous fungi over bacteria for the synthesis of NPs was attributed to its innate characteristics such as high metal tolerance, intracellular metal uptake capability, and cell-wall-binding capacity [128]. Previously, [129] incubated fungus *Aspergillus flavus* with AgNO_3_ solution for 72 h, and it was demonstrated that the AgNPs accumulated on the surface of the cell wall of the fungus. In another study, culture supernatant (CS) [128] obtained from the fungus *Cunninghamella phaeospora* was employed for the creation of AgNPs. The resultant NPs were mostly spherical in shape, having a size of around 12.2 nm. The other characteristics shown by AgNPs included their monodisperse nature and, most importantly, their broad-spectrum antibacterial activity. The fungal species which can be utilized for the biosynthesis of both gold (Au) and silver (Ag) NPs include [108] *Fusarium* sp., *Penicillium* sp., and *Aspergillus* sp. Silver nanoparticles can be synthesized using *Trichoderma viride* fungus [130] via the extracellular biosynthesis method [4]. Stable AgNPs having a size range of 5 to 15 nm can be synthesized using *Fusarium oxysporum*, wherein the NADH-dependent reductase enzyme reduces the silver ions [131]. A recent study presented by [132] explored an in vitro application for the generation of AgNPs using the fungus *Penicillium citrinum*. Similarly to bacteria [91], fungi possess metal tolerance, metal bioaccumulation ability, uptake via an intracellular route, and high binding capacity [91]. In comparison with other microorganisms, fungi are highly beneficial for the synthesis of NPs on an appreciably large scale. For example, as compared to bacteria and plant extracts, the fungal mycelial mesh has a better capacity to tolerate conditions such as pressure, flow, and agitation present in bioreactors and chambers. Fungi secrete various enzymes or several proteins per unit of biomass, which leads to higher yields than the nanoparticles formed in a bacteria-mediated process. Fungi are quick to reproduce and grow. More reductive proteins are secreted via the extracellular route by fungi than bacteria. The extracellular synthesis of NPs is beneficial as resultant NPs are free from redundant cellular components and they do not bind to the biomass; hence, they can be used without further treatment for various purposes [91]. Furthermore, the increased application of fungi in the biosynthesis of NPs in comparison to other microorganisms is attributed to their ease of handling and ecofriendly nature; for example, the nonpathogenic white rot fungus could be utilized for the synthesis of AgNPs on a large scale [133].

#### 2.2.3. Yeast-Mediated Green Synthesis of Silver NPs

Yeasts represent another class of microorganisms explored for the green synthesis of AgNPs [133]. Yeast is a eukaryotic, unicellular organism which has evolved from multicellular antecedents. Yeasts, being chemoorganotrophs, utilize organic compounds such as carbon obtained from sugars as their primary source of energy. They are known to grow well in neutral or slightly acidic environments. Newer methods of cultivation of yeast are devoid of all the exasperating steps, thus resulting in a simpler and easier process [133]. Currently, the focus has shifted from prokaryote-based biosynthesis to eukaryote-mediated green synthesis, which has expanded the scope and broadened the future possibilities for the ecofriendly generation of NPs. In another instance, Korbekandi et al. showed the utilization of AgNPs during biotransformation using *Saccharomyces cerevisiae*, commonly known as brewer’s yeast, wherein the synthesis of NPs took place in various parts of the cells, such as in the core of the cells and the cell membrane, adhering to the cell membrane and possibly the exterior of the yeast cells. Thus, they are preferred to bacteria for NP synthesis because of the potential scale-up, easy handling, and regulation at lab scale using only simple nutrients [134].

#### 2.2.4. Algae-Mediated Synthesis of AgNPs

Algae [133] are a diverse group of aquatic organisms. They are eukaryotic species capable of conducting photosynthesis. Thus, in aquatic atmospheres, they are prevalent primary producers, including cyanobacteria. Algae have a wide distribution of sizes [133], varying from microscopic picoplankton to Rhodophyta, which has a size in the macroscopic range. Algal *Chlorella* sp. was reported to possess the ability to accumulate heavy metals such as uranium [135], cadmium, zinc, nickel, and copper [136]. Algae-mediated biosynthesis [133] has been found to be rapid and inexpensive [108]. The selection of algae as a biological source is attributed to its negatively charged cell surface, which has the capacity to accumulate and grow the crystals rapidly. Its associated low cost also makes mass production of nanoparticles a realizable possibility. In the case of *Chaetomorpha linum* algae, the algal metabolites assist in the reduction of AgNO_3_. The metabolites, essentially terpenoids and flavonoids, aid in the capping and stabilization of nanoparticles. Other metabolites such as polysaccharides are found to be effective in controlling the shape and size of AgNPs [137]. AgNPs can be synthesized using marine algae *Cystophora moniliformis* for the reduction of ions and stabilization of NPs. Patel et al. also stated that AgNPs synthesized by strains of *Coelastrum* sp. and *Botryococcus braunii* exhibited antibacterial activity against tested pathogens such as *B. megaterium*, *E. coli*, *B. subtilis*, *M. luteus*, *P. aeruginosa*, and *S. aureus.* [138]. Other algal extracts used to produce AgNPs include those obtained from red algal seaweeds of *Laurenciella* sp. and *Laurencia aldingensis* [133]. Significant cytotoxicity was observed against uterine sarcoma MESSA/Dx5, as well as its parental MESSA cell line, when subjected to synthesized AgNP. In contrast, no toxicity was observed against P4 (human foreskin fibroblasts) health cells. The study indicated that the AgNPs can be used as potentially novel *Candida*tes for chemotherapy [139].

#### 2.2.5. Actinomycetes-Mediated Green Synthesis of Silver NPs

Actinomycetes belong to the order of Actinobacteria, a phylum of bacteria called Actinomycetales. Actinobacteria are mostly Gram-positive. They may or may not be anaerobic. As discussed earlier, two mechanisms elaborated in microbe-mediated synthesis are intracellular synthesis and extracellular synthesis, and actinomycetes are good *Candida*tes for both types of synthesis [113]. It was found that the cell wall and cell membrane of actinomycetes contain the NADH-dependent reductase enzyme, which is useful to reduce gold and silver ions, and the secreted proteins such as cytochrome C act as capping and stabilizing agents for the synthesized NPs [140]. In one such demonstration [133], *Streptomyces* species were utilized to generate spherical AgNPs of 20–70 nm size range. The actinomycetes-mediated synthesis of NPs yields stable and polydisperse NPs which usually have remarkable antimicrobial capacity [113]. AgNPs synthesized using a culture supernatant of *Streptomyces* sp. JAR1 showed significant inhibitory activity toward pathogens such as *Candida tropicalis*, *Salmonella* spp., *E. coli*, *Scedosporium* spp., *Pseudomonas aeruginosa*, *Ganoderma* spp., *Fusarium* spp., and *Staphylococcus* aureus [141].

### 2.3. Enzyme-Based Synthesis of Silver NPs

The purity of available enzymes [4] and their structure make this synthetic route a prospective method to create silver nanoparticles of the desired form. The extracellular synthesis of AgNPs is attributed to the enzymes released by the cells [91]. The enzymes present in the extracts of plants could also act as a reducing agent in this green AgNP synthesis. During the enzyme-based synthesis of AgNPs, a specific enzyme is obtained from the cultural supernatant of lifeforms such as bacteria. Kumar et al. (2007) first demonstrated the in vitro fabrication of AgNPs using the α-NADPH-dependent nitrate reductase enzyme obtained from the cultural supernatant of fungus *Fusarium oxysporum* and phytochelatin [142].

#### Biomolecule-Mediated Synthesis of Silver NPs

The biosynthesis of AgNPs [15] has been explored using monosaccharides such as glucose, fructose, and galactose several disaccharides such as lactose and maltose, and numerous polysaccharide molecules such as starch, heparin, dextran, chitosan, and pectin. There are convincing studies available on utilizing starch as a stabilizing and reducing agent in this process of synthesizing nanoparticles. Hence, polysaccharides serve as a reducing and a capping agent. In one of the experiments, the in situ method of preparation was utilized for the preparation of green silver nanocomposites. In this experiment, a colloidal silver dispersion was prepared by employing glucose as a reducing agent, water as a solvent, and soluble starch as a stabilizing agent. When the produced green AgNPs were dispersed in a potato starch/glycerol matrix, the process yielded silver nanocomposites with potential application in antimicrobial packaging [143]. According to an experiment performed by Zain et al. (2014), when ascorbic acid was utilized as a reducing agent in chitosan solution for the creation of AgNPs and CuNPs (copper nanoparticles) using microwave heating, the resultant AgNPs were found to possess greater bactericidal activity against bacterial species such as *Bacillus subtilis* and *E. coli* as compared to CuNPs of the same mean size [144]. Another example is represented by levan, which is a polysaccharide mainly derived from plants but is also obtained from microorganisms and curdlan (a bacterial exopolysaccharide), which have all been employed to fabricate appreciably stable AgNPs. The particles synthesized using curdlan as a reducing and stabilizing agent were observed to be mostly spherical in shape with a mean diameter around 15 nm [145]. Furthermore, vitamins and amino acids have proven their suitability, especially for the synthesis of therapeutic nanoparticles [15]. The literature has also mentioned the capping and reducing properties of vitamin B2 for the synthesis of silver and palladium (Pd) nanoparticles. The NPs were found to self-assemble into different shapes depending on the solvent used for their preparation. The AgNPs and PdNPs were found to self-assemble into structures of nanorods and nanowires when solvents such as water and isopropanol were used, respectively. These self-assemblies of AgNPs and PdNPs further catalyzed the reactions of pyrrole and aniline to form polypyrrole and polyaniline nanocomposites [146]. Furthermore, glycerol has recently been considered as a promising green solvent in the synthesis of various metallic NPs. Because of its low toxicity, glycerol is preferred as a cheaper and better alternative to usually employed polyols, including propylene glycol and ethylene glycol [147]. For example, the process of synthesizing AgNPs was accomplished using starch as a capping agent and β-d-glucose as a reducing agent. The resultant nanoparticles were found to be stable and similar in properties (e.g., polydispersity and shape) to AgNPs obtained using conventional techniques of synthesis [148]. AgNP [24] synthesis utilizes water (solvent) and polysaccharides (capping agent) such as starch and heparin in this process. This method is reported to be advantageous as the binding interactions between AgNPs and starch are quite weak and are noted to reverse at higher temperatures, thus assisting the segregation of the obtained NPs. Additionally, surface passivation and a significant prevention of particle aggregation occur due to the extensive hydrogen bonding network [148].

Furthermore, the literature highlighted a synthesis pathway that employs negatively charged heparin as a reducing and stabilizing agent in a mixture of AgNO_3_ and heparin heated to 70 °C for approximately 8 h [120]. The anionic nature of the sulfonate groups present in heparin facilitates the formation of silver nanoparticles. There is another strategy known as the Tollens method that employs a single-step process to form AgNPs of dictated size [149,150]. In an altered Tollens procedure, positively charged silver ions are reduced by sugars such as fructose, glucose, xylose, and maltose in the presence of ammonia. This procedure yields AgNPs of various shapes with a particle size ranging from 50–200 nm. Furthermore, investigations also revealed that the smallest particle size was obtained at the lowest ammonia concentration [151]. Some scientists also mentioned the autoclave technique for producing AgNPs using starch, whereby, similarly to previous examples, starch acts as a reducing and stabilizing agent [152,153].

### 2.4. Green Synthesis from Vitamins

The synthesis of gold and palladium nanospheres, nanowires, or nanorods has been reported, wherein vitamin B2 was employed as a reducing and capping agent. Vitamin B2 employed here acts as a reducing agent in the process of synthesis. Such a synthesis approach can be further extended to form silver nanostructures. Ascorbic acid or vitamin C has also been employed as a capping and reducing agent, whereas chitosan is used as a stabilizing agent because it binds to charged metal species [154]. An interesting process for the synthesis of NPs possessing uniform size was developed using ascorbic acid [155]. Even during glycolysis, plants produce H^+^ ions along with NAD, which play the role of strong reducing agents in the synthesis of AgNPs [156].

### 2.5. Ionic Liquid-Mediated Synthesis

Ionic liquids (ILs) have recently emerged as a new option of reaction medium [157] due to their appreciable properties such as low volatility, nonflammability, high chemical and thermal stabilities, designable structures, high ionic conductivity, and broad electrochemical windows. Hence, ionic liquid-mediated [158] synthesis is gaining preference in the green chemistry world through their use as green electrolytes. Chemical and electrochemical AgNP synthesis utilizing a similar method was developed for the synthesis of AgX (X = Cl, I) NPs by employing ionic liquids [159]. In another study, ionic liquids of bis(alkylethylenediamine) silver(I)) salts such as bis(*N*-2-ethylhexylethylene diamine) silver (I) nitrate and bis(*N*-hexylethylenediamine) silver (I) hexafluorophosphate were used for the synthesis of AgNPs. It was found that uniform AgNPs were formed successfully via the reduction of bis(N-2-ethylhexylethylenediamine) silver (I) nitrate solution with aqueous NaBH_4_ but not by the reduction of bis(*N*-hexylethylenediamine) silver (I) hexafluorophosphate [160]. Pringle et al. (2008) demonstrated a single-step process of conducting polymer–AgNP composite synthesis using an ionic liquid solution of silver nitrate. The ionic liquid used for the experiment was 1-ethyl-3-methylimidazolium bis(trifluoromethanesulfonyl)amide [161]. In addition, synthesis of an Ag–carbon hybrid with controlled structure and morphology via a hydrothermal treatment of silver nitrate and glucose was achieved using ionic liquid tetradecyl-3-methylimidazolium tetrafluoroborate as a soft template [162], whereas the synthesis of partially positively charged AgNPs was also achieved using ionic liquid 1-butyl-3-methylimidazolium tetrafluoroborate [163]. In one of the experiments, the electrodeposition of silver from the distillable ionic liquid DIMCARB, formed by mixing CO_2_ and Me_2_NH in a specific proportion, was carried out [164]. Furthermore, the fabrication of spherical and polygonal AgNPs in ionic liquid bmimBF_4_ using an electrochemical method was reported. In this case, polyvinylchloride (PVP) was used as the stabilizer. The study revealed that, in comparison with water which is a traditional solvent, ionic liquids offer many advantages including a shortened electrolytic time, reduced PVP dosage, and reduced energy consumption, as the process allows electrolysis without the requirement of mechanical stirring [165].

### 2.6. Irradiation-Assisted Synthesis

According to the sixth principle of green chemistry, i.e., design for energy efficiency, irradiation-assisted synthesis is an excellent green method [166]. Irradiation-assisted synthesis does not require the use of additional reducing agents, thus avoiding the associated side reactions and toxicity (if any). Accordingly, this method of synthesis also obeys other principles of green chemistry. A number of irradiation methods including laser irradiation, radiolysis, and pulse radiolysis can be employed for the synthesis of AgNPs [1]. AgNPs can be fabricated with a distinct size and shape via laser irradiation [1] of an aqueous solution of silver salt and a surfactant solution of sodium dodecyl sulfate (SDS) [167]. In another instance, a laser was utilized in a photo-sensitization method for AgNP formation by employing benzophenone [168]. AgNPs of around 20 nm were obtained by employing low-power lasers for short irradiation times. However, NPs 5 nm in size were formed when a greater irradiation power was supplied. They were also successfully synthesized via this concept using a mercury lamp [168]. The photosensitized growth of AgNPs was also demonstrated in visible-light irradiation studies, wherein thiophene was employed as a sensitizing dye [169], and AgNP synthesis was completed via the illumination of Ag(NH_3_)^+^ in ethanol [170]. Huang et al. (2009) synthesized green AgNPs using a chitosan aqueous solution as the stabilizing agent under γ-irradiation without isopropanol and an N_2_ atmosphere. The minimum inhibitory concentration (MIC) value of the synthesized AgNPs was found to be around 100 ppm when tested against methicillin-resistant *Staphylococcus aureus* and *Aeromonas hydrophila* for their antibacterial efficacy. The resultant AgNPs were found to exhibit good antibacterial activity [171].

### 2.7. Microwave-Assisted Synthesis

Uniform AgNPs were also fabricated by microwave radiation [1,24] of a carboxymethyl cellulose sodium (CMS) and silver nitrate solution. The NPs generated were uniform and appreciably stable at room temperature for around 2 months [172]. CMS can be employed as both a reducing and a stabilizing agent in the process of formation of AgNPs [172]. Moreover, only AgNO_3_ is required in the reaction as a reagent. When compared to the general heating treatment [4], this method provides homogeneous heating and appreciable nucleation of noble-metal NPs in a simple manner [173]. It was employed as a fast process for NP formation (a matter of seconds) using irradiation at 50 W [174], wherein red grape pomace was employed as a reducing agent. Furthermore, microwave radiation was applied to create monodisperse AgNPs by employing l-lysine or l-arginine and soluble starch as reducing and protecting agents, respectively [175]. Noroozi et al. (2012) achieved rapid microwave-mediated green synthesis of AgNPs without using a reducing agent. AgNPs were produced by using polyvinylpyrrolidone (PVP) as the stabilizing agent in a water medium. In comparison to silver nanoparticles produced using the conventional heating method, the AgNPs fabricated using microwave irradiation were found to be denser, more uniform, and smaller in size. These AgNPs can be used to formulate size-dependent nanomedicines [176]. While analyzing the microwave-assisted synthesis of green AgNPs using peel extracts of citrus fruits, Kahrilas et al. realized that silver nanoparticles were successfully produced only when orange peel extract was subjected to synthesis, where it worked as a reducing and capping agent. Thus, the study showed a greener alternative to toxic reducing and capping agents [177]. Later, AgNP creation using biomaterials such as sodium alginate was also reported and, interestingly, the synthesized spherical nanoparticles were noted to be stable for about 6 months or more when stored at room temperature. The AgNPs also showed good antibacterial activity toward *Escherichia coli* and *Staphylococcus aureus* [178]. In another study, Albadran and Kamal et al. worked on the optimization and modeling of the green synthesis of AgNPs by employing a one-pot microwave-mediated method. In this study, a microwave-assisted reaction of AgNO_3_ and cactus extract was carried out to produce a colloidal suspension of AgNPs. The resultant colloid suspension of AgNPs was further investigated for its absorbance and photocatalytic activity in the removal of organic pollutants from wastewater [179]. Anjana et al. (2021) demonstrated the rapid microwave-assisted synthesis of stable AgNPs by employing leaf extracts of *Cyanthillium cinereum*. The resultant AgNPs were found to demonstrate antibacterial activity toward bacterial species such as *Klebsiella pneumoniae* and *Staphylococcus aureus*. In addition, it was suggested that the resultant nanoparticles can be employed as promising *Candida*tes in applications as biosensors and nonenzymatic electrochemical sensors [180].

## 3. Characterization of Green Silver Nanoparticles

Silver nanoparticles are typically characterized [128] according to their size, surface charge, distribution, surface morphology (shape), aggregation, etc. The physicochemical properties have a notable impact on the biological properties; thus, physicochemical characterization of nanoparticles is typically conducted prior to in vitro and in vivo studies [181]. It has also been stated that numerous physiochemical properties can particularly impact the interactions between the nanomaterials and specific target sites (proteins, cells) [182]. To evaluate these properties, a number of analytical techniques are utilized such as ultraviolet/visible spectroscopy, X-ray diffractometry (XRD), Fourier-transform infrared spectroscopy (FTIR), scanning electron microscopy (SEM), dynamic light scattering (DLS), atomic force microscopy (AFM), transmission electron microscopy (TEM), and zeta potential analysis [183].

### 3.1. Surface Morphology

SEM and TEM are the two main types of electron microscopy. TEM is popularly used for the characterization of nanomaterials. TEM is used to determine the size, degree of aggregation, dispersion, etc. in nanomaterials. SEM is employed in addition to TEM for the structural analysis of NPs. Compared to SEM, TEM is considered more advantageous as it provides good-quality spatial resolution along with precise analytical measurements. For instance, SEM and TEM were used to clarify the morphology and size of the resultant NPs in [4]. SEM analysis has mostly revealed that the AgNPs formed in the literature were spherical, although a few authors reported irregular [184], triangular [185], hexagonal [129], isotropic [186], polyhedral [187], flake [188], flower [189], pentagonal [190], anisotropic [191], and rod-like structures [192]. Zeta potential values are used to confer colloidal stability to the integrated NPs, allowing the solution or dispersion to resist aggregation [193]. Scientists have employed macroalga *Spirogyra varians* to synthesize NPs with an average size of 35 nm in uniform and quasi-spherical shapes [37]. Abd-Elnaby et al. characterized AgNPs synthesized extracellularly from marine *Streptomyces rochei* MHM13, and SEM confirmed the spherical shape of AgNPs 22 to 85 nm in size [44]. Singh et al. reported the TEM results of AgNPs synthesized using an endophytic fungal supernatant of *Raphanus sativus*, whereby they observed that AgNPs were almost spherical with a size ranging from 10 nm to 30 nm [49]. In another experiment, *W. oryzae* DC6-mediated AgNPs, when studied by FE-TEM, showed a spherical shape with a 10–30 nm size range [53]. Suresh et al. studied AgNPs synthesized using *Delphinium denudatum* root extract using FE-SEM. The synthesized AgNPs were found to be polydisperse and spherical with a size mostly below 85 nm [56].

Atomic force microscopy (AFM) is a scanning probe microscopy (SPM) method with greater resolution, and it is capable of creating 3D images of surfaces at high magnification [194]. When AFM was used for the determination of size and morphology of green AgNPs biosynthesized using an endophytic fungal supernatant of *Raphanus sativus*, the study showed the presence of monodisperse AgNPs having an average particle size of 4 to 28 nm [49]. Praphulla Rao et al. synthesized AgNPs using lemon extract, and further characterization using AFM suggested that the NPs were nearly 12 nm height and 100 nm in width [62]. Bankura et al. presented an AFM image of dextran-mediated AgNPs, showing the formation of well-dispersed NPs in the 10 nm to 60 nm size range [64]. Minhas et al. evaluated the surface properties of biogenic AgNPs synthesized using extracts of *Ulva compressa* L. Kütz. and *Cladophora glomerata* L. Kütz., wherein characterization of NPs using AFM revealed the roughness parameters average roughness (Sa), root-mean-square roughness (Sq), and ten-point height (Sz). For AgNPs synthesized using an extract of *U. compressa*, the measurements obtained were as follows: Sa, 1.01 nm; Sq, 1.48 nm; Sz, 9.09 nm, whereas, for AgNPs synthesized using an extract of *C. glomerata*, the values obtained were as follows: Sa, 0.471 nm; Sq, 0.848 nm; Sz, 5.90 nm [36]. Gopinath et al. utilized the dried fruit body extract of *Tribulus terrestris* for the synthesis of AgNPs. The AFM study revealed the formation of spherical AgNPs that were mostly homogeneous in size with individual particles in the range of 24.631 nm [61].

### 3.2. UV/Visible Spectroscopy

UV/visible spectra assist in analyzing the relationship between the metal ion concentration, pH, and extract content and the type of AgNPs formed. The emergence of a yellow or light brown-yellow color in a previously colorless mixture is usually indicative of the formation of AgNPs. The optical properties also depend on the particle size and shape [195].

The characteristic peak of AgNPs in a UV/visible spectrum is usually observed around 430 nm; however, as discussed earlier, many factors affect the position of this peak. In the green synthesis of AgNPs using macroalgae *Spirogyra varians* [37], *Streptomyces rochei* MHM13 [44], the endophytic fungal supernatant of *Raphanus sativus* [49], *Weissella oryzae* DC6 [1], *Delphinium denudatum* root [56], *Piper nigrum* leaf and stem [59], and dextran [64], the absorption peaks in the UV/visible spectrum were observed at 430 nm, 410 nm, 426 nm, 432 nm, 416 nm, 460 nm, and 423 nm, respectively.

### 3.3. SAED

Selected area (electron) diffraction (SAED) is a diffraction technique used to determine the crystal structure of NPs. Studies are usually conducted in a TEM or SEM using electron backscatter diffraction [194]. AgNPs are generally crystalline in nature having an fcc (face-centered cubic) structure. During the characterization of AgNPs synthesized using an endophytic fungal supernatant of *Raphanus sativus*, Singh et al. found bright rings in the SAED pattern, indicating the crystalline nature of NPs [49]. Similar results were obtained in case of green AgNPs synthesized using marine endophytic fungus *Penicillium polonicum* [51]. In another study, Bankura et al. synthesized AgNPs using dextran; during SAED analysis, circular rings corresponding to the (111), (200), (220), (211), and (222) planes were observed in the SAED pattern, indicating the highly crystalline nature of resultant AgNPs [64]. Additionally, the SAED pattern of AgNPs synthesized using gum kondagogu (*Cochlospermum gossypium*) consisted of concentric rings corresponding to the (111), (200), (220), and (311) planes of face-centered cubic (fcc) silver with intermittent bright dots, revealing the highly crystalline nature of these NPs [63].

### 3.4. XRD

XRD studies are employed to determine the crystal structure of nanomaterials. They are also used to record the size and shape of the unit cell from the peak points via translational symmetry [196]. This technique is basically employed for the analysis of atomic spacing and crystal structures. The observations are based on the constructive interference of a crystalline sample and the monochromatic X-rays generated by a cathode ray tube, which are then filtered to produce monochromatic radiations. This method aids to understand the crystal structure and atomic properties of the synthesized AgNPs, along with their size measurements [197].

In a synthesis study of AgNPs using macroalgae *Spirogyra varians*, four different and important characteristic peaks were observed after 2 h at 38.1°, 44.3°, 64.5°, and 76.4° corresponding to the (111), (200), (220), and (311) planes, respectively. All peaks in the XRD pattern can be readily indexed to the face-centered cubic structure of silver [37]. A similar trend was observed in case of green AgNPs synthesized using an endophytic fungal supernatant of *Raphanus sativus* [49], *Piper nigrum* leaf and stem [59], and dextran [64]. Singh et al. analyzed *Weissella oryzae* DC6-mediated AgNPs using XRD, showing that the XRD pattern of fabricated NPs showed extreme peaks across the whole spectrum of 2θ values ranging from 20–80, and this pattern was analogous to the Braggs reflection of silver nanocrystals [53]. Suresh et al. observed 13 intense peaks (12.25°, 13.12°, 16.07°, 16.68°, 18.60°, 20.30°, 27.24°, 27.63°, 28.30°, 30.31°, 30.89°, 38.45°, and 44.87°) across the whole spectrum of a *Delphinium denudatum* root extract after 2 h, with values ranging from 10 to 70, indicating the presence of both cubic and fcc structured NPs, i.e., a mixed phase of AgNPs. In another study, Shameli et al. prepared PEG-based AgNPs via a green synthesis route and characterized them using XRD. The results indicates peaks at 38.04°, 44.08°, 64.36°, and 77.22° attributed to the crystallinity of the AgNPs [198].

### 3.5. FTIR

**Figure 6:**
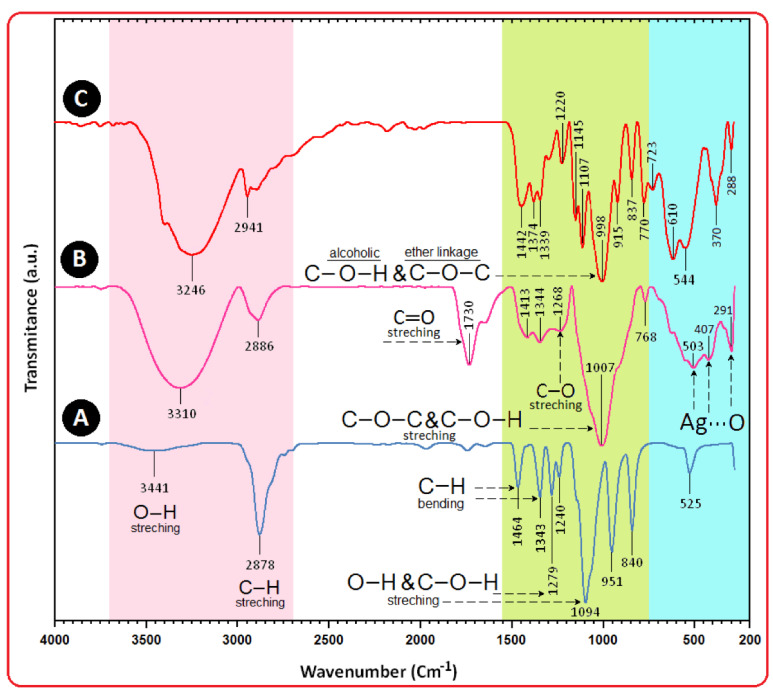
FTIR spectra for PEG (**A**), [Ag(PEG)] for the stirring time of 48 h (**B**) and β-D-glucose (**C**). Figure was adopted from [198]; CC-BY License.

FTIR is used to analyze the functional groups or metabolites (capping/stabilizing agents) employed on the surface of NPs [128]. Salari et al. reported the presence of amino, carboxylic, hydroxyl, and carbonyl groups following an FTIR study of AgNPs synthesized using macroalgae *Spirogyra varians*. The FTIR spectra displayed several peaks attributed to different functional groups including strong broad O–H stretching carboxylic bands around 3423 cm^−1^, carboxylic/phenolic stretching bands around 2927 cm^−1^, and quinine OH bands around 1515 and 1429 cm^−1^. After the synthesis of AgNPs, the characteristics peaks of proteins appearing around 1645 cm^−1^ were found to be shifted [37]. In the FTIR spectrum of AgNPs synthesized using marine *Streptomyces rochei* MHM13, an array of absorbance bands were observed from 400 to 4000 cm^−1^. Characteristic intense peaks appeared at 3420.14, 2932.23, 2362.37, 1639.20, 1430.92, 1115.62, and 613.252 cm^−1^, which denoted the presence of capping and stabilizing agents containing several functional groups [44]. The FTIR spectrum of AgNPs synthesized using an endophytic fungal supernatant of *Raphanus sativus* displayed absorption bands at 3145, 1597, 1402, 1109, 1213, 995, 911, 699, and 504 cm^−1^. These peaks were attributed to several functional groups such as hydroxyl, amino, amino acid, methyl, alkene, and alkyl halides [49]. Suresh et al. observed FTIR peaks at 3354, 2952, 2063, 1651, 1419, 1383, 1354, 1171, 1093, 780, 672, and 605 cm^−1^ for AgNPs synthesized using *Delphinium denudatum* root extract. Comparing the peaks of the spectrum of synthesized AgNPs and *Delphinium denudatum* root extract, they concluded that the polyols and phenols acted as reducing agents, whereas some proteins and metabolites (e.g., terpenoids) with functional groups such as amine, alcohol, ketone, aldehyde, and carboxylic acid acted as capping and stabilizing agents [56]. During the FTIR analysis of AgNPs synthesized using a leaf extract of *P. nigrum*, absorption bands were observed at 3314, 3197, 2897, 2362, 1763, 1668, 1628, 1532, 1480, 1399, 1383, 1335, 1276, 1191, 1122, 884, 823, 750 cm, 656, and 602 cm^−1^, indicating that the phytochemicals responsible for the synthesis of NPs contained functional groups such as amino, alkyl, carboxyl, carbonyl, nitro, alkyne, and ester. In the case of stem-derived AgNPs, the bands observed at 3697, 3313, 3195, 2298, 1456, 1336, 1670, 1193, 1118, 811, 750, 651, and 601 cm^−1^ allowed concluding that the phytochemicals contained functional groups including amino, nitrile, amide, ester, and alkynes [59]. Furthermore, Shameli et al. prepared PEG-based AgNPs via a green synthesis route and characterized them using FTIR; the interaction between AgNPs and PEG molecules was established by the peak at 1730 cm^−1^ (–C=O carboxylic acid group in gluconic acid) and a shift in the peak at 1007 cm^−1^ toward a lower frequency (Figure 6A–C) [198].

## 4. Antimicrobial Applications of Green Silver Nanoparticles

### 4.1. Antibacterial Applications

It is well known that elemental silver and its various compounds [30] have been utilized for decades to preserve water in the form of silver coins/silver vessels. Since the Middle Ages [199], silver has been used as an inhibitory and antibacterial material, highlighting its activity as an antibacterial agent. The antimicrobial properties of AgNPs obtained via green synthesis are summarized in Table 1. In one study, it was proven that AgNPs [199] synthesized by employing black cohosh, geranium, aloe, etc. at a concentration of around 4 ppm exhibited an inhibitory effect on the proliferation of *E. coli*. The NPs synthesized using aloe displayed the highest antibacterial activity, whereas the black cohosh and geranium NPs did not display a notable effect. The aloe-based NPs displayed greater activity toward the growth of *Salmonella typhimurium* than that of *E. coli* [200]. These plant extract-based nanoparticles exhibited greater inhibitory effects against *Salmonella* and *E. coli*, whereas they exerted moderate activity toward *Pseudomonas aeruginosa*, *Kocura rhizophila*, and *Bacillus subtilis*. Some scientists have proposed that AgNPs can show significant activity toward our respiratory system and cell membrane permeability by adhering to their surface [58]. Furthermore, it has been observed that AgNPs can perforate the bacterial cell membrane surface. Moreover, they have also been found to exert greater activity toward Gram-negative bacteria than Gram-positive bacteria [58]. For instance, in one report, AgNPs synthesized using *Aloe vera* via a hydrothermal method were studied for their bactericidal effect against pathogenic Gram-positive *S. epidermidis* and Gram-negative *P. aeruginosa*, wherein, at a lower AgNP concentration, there was major lethality exhibited toward Gram-negative bacteria only, whereas higher concentrations also displayed activity toward Gram-positive bacteria [58]. This is possibly because of the beta barrel proteins present, which are commonly known as porins, as well as the thinner peptidoglycan layer in Gram-negative bacteria compared to Gram-positive bacteria. However, it has also been observed that, when the surface area of NPs is increased, the surface energy also increases, thereby increasing their effectiveness [83]. Therefore, even at a lower concentration, smaller NPs possessing a greater surface-area-to-volume ratio exhibit significant antibacterial activity. It was also observed in a report that Gram-negative bacterium *E. coli* displayed a maximum zone of inhibition of 10.75 mm, possibly due to the cell wall of Gram-positive bacteria being composed of a thick peptidoglycan layer forming a rigid structure, leading to difficult penetration of the AgNPs, unlike Gram-negative bacteria, whose cell wall constitutes a thinner peptidoglycan layer. The high bactericidal activity is certainly attributed to the silver cations released from AgNPs acting as reservoirs of Ag^+^ (bactericidal agent) ions. These ions from the NPs are speculated to attach to the negatively charged bacterial cell wall and rupture it, leading to protein denaturation and cell death [61].

Several studies support the appreciable antibacterial activity displayed by various green AgNPs. For instance, a study evaluating the activity of Fu-AgNPs toward *K. pneumoniae*, a Gram-negative bacterium which causes *Klebsiella* infections, revealed that the antibacterial activity of these AgNPs was impressive by inhibiting the multiplication of bacterial cells despite their multidrug resistance [37]. In another report, the disc diffusion method was utilized to evaluate the antibacterial activity of AgNPs fabricated from *S. fusiformis* toward human bacterial pathogens such as *E. coli*, *S. aureus*, *K. pneumoniae*, and *P. aeruginosa*, wherein the zone of inhibition was measured to obtain results in line with the proposed characteristic activity of the AgNPs [39]. The results of another experiment indicated that the extracellularly fabricated AgNPs employing *P. deceptionensis* DC5 revealed the highest antimicrobial potential against *V. parahaemolyticus*, followed by *C. albicans*, *S. aureus*, *S. enterica*, and *B. anthracis*.

A well-diffusion method revealed that bio(AgNPs) exhibited the highest antimicrobial activity toward *P. aeruginosa*, *S. aureus*, and *P. mirabilis* (10 mm for all), followed by *E. coli*, *K. pneumoniae*, and *B. subtilis* (6 mm for all). However, no activity was detected toward *S. infantis*. The authors also observed synergistic effects of the green AgNPs with antibiotics against bacterial pathogens. For instance, the antibacterial effect of ampicillin, kanamycin, and tetracycline was enriched in the presence of bio(AgNPs) against *S. aureus*, *K. pneumoniae*, and *P. aeruginosa*, along with improved streptomycin activity toward *P. mirabilis* when combined with these AgNPs [43] (see Table 1).

In line with these results, numerous bacterial pathogens exhibit distinct susceptibilities to AgNPs. A study was conducted on the combined effect of AgNPs with six standard antibiotic discs (ciprofloxacin, streptomycin, ampicillin, tetracycline, gentamicin, and lincomycin) against a few multidrug-resistant pathogenic bacteria [201]. For all tested cases, the resulting inhibition zone diameters were notably increased when the antibiotics were combined with AgNPs. It is, thus, evident that the synergy of AgNPs with antibiotics has appreciable antimicrobial effects, which hinders the development of resistance and improves the antimicrobial properties of the antibiotics, while also decreasing their dosage [44]. In addition to their antibacterial activity, the cytotoxic activity of AgNPs on Hep2 cell lines was also studied using the method of Daikoku et al. (1989); moreover, on similar lines, [40] first reported the cytotoxic activity of AgNPs synthesized using seaweed extracts of *G. corticate*. Moreover, nanoparticles are well known for their target specificity. In one study, silver nanoparticles were synthesized using *B. methylotrophicus* DC3 via an ecofriendly approach, showing antimicrobial activity toward numerous pathogenic microorganisms. Several metals and their respective salts [78] have been reported to possess antibacterial activity toward *H. pylori* [202]. The activity of these metallic agents may be due to the inactivation of *H. pylori* urease [203]. Even particles synthesized via olive leaf extract [78] showed significant antibacterial activities. In another instance, silver nanoparticles synthesized using [204] carob leaf extract displayed significant activity toward *E. coli*. The synthesized nanoparticles also exhibited significant inhibition activity toward *C. albicans*.

AgNPs synthesized [158] using *Spirogyra varians* were found to be 17.6 nm in size, and they acted as an appreciable antibacterial agent. These particles also offered potential effects against [103] multidrug-susceptible and -resistant strains such as ampicillin-resistant *Escherichia coli*, *Pseudomonas aeruginosa*, methicillin-resistant *Staphylococcus aureus* (MRSA), erythromycin-resistant *Streptococcus pyogenes*, and vancomycin-resistant *Staphylococcus aureus* (VRSA). Their antimicrobial activities [92] are also represented by the widespread use of AgNPs in cardiovascular implants. Interestingly, a prosthetic silicone heart valve was the first cardiovascular device to be coated with silver [92]. Here, Ag was employed to prevent bacterial contamination from taking place on the silicone valve. This further reduced the inflammation of the heart due to contamination. However, it was found that silver induces an allergic reaction and inhibits normal fibroblast function, among other effects, in patients during clinical trials. Consequently, the incorporation of AgNPs in medical devices may provide a nontoxic, safe, and antibacterial coating to overcome the stated challenges.

Another very prevalent use of AgNPs is for catheters in hospitals, which show a significant probability of contamination, thus leading to further complications. In one study, the synthesized AgNPs were examined for the inhibition of biofilm formation by *S. aureus* and *P. aeruginosa*. It was observed that a 5–6 μg concentration of AgNPs was sufficient for the inhibition of biofilm formation by these microbes. Therefore, the synthesized NPs clearly presented appreciable antimicrobial and biofilm inhibition potential, suggesting potential application as antimicrobial agents in the future [53]. For instance, polyurethane catheters are coated with AgNPs to create antibacterial catheters. It was observed that such modified catheters can significantly reduce the growth of bacteria up to 72 h in several models involving animals. Silver injury dressings are utilized to treat various injuries, chronic ulcers, burns, toxic epidermal necrolysis, etc. Furthermore, AgNPs utilized in wound dressing reduce the therapeutic time of injury by a standard of 3.35 days and prevent bacterial growth. They do not have any adverse impacts on patients when compared to standard gauze dressing and Ag sulfadiazine. Another study used chitosan AgNPs for dressing wounds, which displayed enhanced therapeutic action compared to 1% Ag sulfadiazine [92].

Many types of orthopedic and orthodontic implants commonly suffer from contamination, leading to further complications in treatment; therefore, AgNPs have also been introduced into plain poly bones to reduce bacterial resistance. They can also aid in decreasing the microbial colonization of coating materials in dentistry and improve antifungal activity. Moreover, AgNPs incorporated into endodontic fillings displayed prolonged activity toward *Staphylococcus aureus*, *Streptococcus milleri*, and *Enterococcus faecalis* [92]. Moreover, the utilization of antimicrobial plant extracts as reducing and capping agents also facilitates the synthesis of nanoparticles possessing greater activity [103]. Thus, if the capping agents themselves have antimicrobial activity, the antimicrobial activity may be further enhanced. The antimicrobial effect of silver nanoparticles is mediated by several factors which should be considered. One important property is the size. In a broader sense, nanoparticles should be smaller than 50 nm to display appreciable antimicrobial activity, while NPs 10–15 nm in size exhibit higher activity. The highest antibacterial activity in the literature was reported [103] in nanoparticles possessing a size of around 5 nm, wherein increased membrane permeability was observed due to the adherence of the NPs onto the cell membrane, further leading to membrane destruction and cell demise [205]. It was stated that AgNPs with an approximate size of 20–80 nm showed toxicity due to Ag^+^ ions being released, whereas those 10 nm in size or smaller displayed better cell–particle interactions and displayed greater toxicity, resulting in increased intracellular bioavailability. Another important factor is the shape. The antimicrobial activity of triangular, spherical, and hexagonal AgNPs toward Gram-negative *E. coli* was compared [206]. It was found that hexagonal NPs displayed the greatest activity, whereas triangular AgNPs showed no activity. Thus, their activity may also depend on their shape. For instance, truncated triangular silver nanoplates displayed the greatest antibacterial activity due to their crystallographic surface structures and larger surface-area-to-volume ratios [207]. A few scientists have stated that the antimicrobial activity of NPs does not depend on their shape; thus, the exact mechanism of this dependency is still unknown.

Another considerable factor is the nanoparticle concentration. This can be directly correlated to microbial species [208]. The zeta potential affects the activity of NPs because electrostatic adhesion affects the interplay between particles and the cell membrane. The nanoparticle surface charge displays a direct relationship with the antibacterial activity [209]. For instance, Gram-positive *Bacillus* strains exhibited lower susceptibility to AgNPs compared to Gram-negative ones due to the repulsion between the negatively charged functional groups of biomolecules present on the cell surface and the negatively charged surface of the nanoparticles.

AgNPs [103] have displayed significant antimicrobial activity alone and in combination with antibiotics, mediated by three possible mechanisms: (i) cell wall and membrane damage, (ii) intracellular penetration and damage, and (iii) oxidative stress [83,103,210]. In one study [24], it was shown that AgNPs formed using disaccharides such as maltose and lactose possessed greater activity compared to those synthesized using monosaccharides such as glucose and galactose. This could be due to the smaller size of disaccharides compared to monosaccharides. The antibacterial effects of these nanoparticles may also correspond to [82] a dual mechanism that includes the bactericidal effect of Ag^+^ ions and the membrane-disrupting effect of the polymer subunits. Moreover, in other applications [24], silver aerosol NPs were found to be significantly active as antimicrobials toward *B. subtilis* [211]. They have also been utilized in antimicrobial water filters, in activated carbon fiber (ACF) filters [212], and for the removal of bioaerosols. AgNPs and AuNPs synthesized extracellularly using *Fusarium oxysporum* have been employed in sterile clothes for use in hospitals to reduce infections by pathogenic bacteria such as *Staphylococcus aureus.* Thus, it is interesting to that AgNPs may exert better antimicrobial activity than standard antibiotics.

### 4.2. Antifungal Applications

It is known that there is a high rate of mortality due to fungal infections [102], which continues to increase due to the limited number of new antifungal targets and the development of prophylactic antifungals leading to the emergence of resistant strains. Antifungal drugs can be adsorbed onto the surface of biogenic silver, which is thought to facilitate antifungal drug delivery [213,214]. Green AgNPs [214] also exhibit antifungal activity because of their bio-coating activity complementing their advantageous size. Green synthesized AgNPs were found to display high antifungal activity toward *Cryptococcus* and *Candida* species. AgNPs [215] formed using *Pilimelia columellifera* subsp. *pallida* SL19 showed activity toward fungi responsible for superficial mycoses, i.e., *M. furfur* and *C. albicans*. Silver nanoparticles synthesized using *Mentha pulegium* [216] aqueous extract displayed significant activity toward fluconazole-resistant *Candida albicans*. Moreover, the colonies of plant pathogenic fungi such as *Magnaporthe grisea* and *Bipolaris sorokiniana* were restrained by nano-ionic silver in vitro conditions [217]. AgNPs were also found to be appreciably active as a broad-spectrum fungicide toward *Botrytis cinerea* and *Alternaria alternata*. They also possess significant collaborative activity with several fungicides toward numerous plant pathogenic fungi such as *Fusarium oxysporum* (tomato wilt) and *Penicillium expansum* (apple rot) [218]. Furthermore, disruption of cell membrane formation [83] and further stoppage of fungal reproduction in *C. albanicans* species were reportedly due to the action of AgNPs [219].

One study investigated the antifungal activity of AgNPs toward 10 fungal pathogens, including *Aspergillus* spp., *Candida* spp., and *Fusarium* spp., revealing significant antifungal activity in all cases. In addition, yeast is also one of the main causes of fungal diseases. In this study, the effect of AgNPs on the growth of yeast was investigated by adding AgNPs to YEPD. The growth curves of *Candida (C. albicans*, *C. parapsilosis*, *C. krusei*, and *C. tropicalis)* in the presence of AgNPs revealed that their growth can be completely inhibited by AgNPs acting as fungistatic agents. There is great scope for further research on a profitable alternative to treat various fungal diseases in the future [47].

### 4.3. Antiparasitic Applications

AgNPs were observed to have larvicidal activity toward *Culex quinquefasciatus* [74], dengue vector *Aedes aegypti* [56], malarial vector *A. subpictus* [190], filariasis vector *C. quinquefasciatus* [220], *Aedes aegypti* [221], *A. subpictu* [220], and other parasites. Although the proper mechanism is not yet known, the denaturation of sulfur-containing proteins and phosphorus-containing DNA by AgNPs further leads to the denaturation of enzymes, organelles, etc., which may be responsible for its activity [222]. Leishmaniasis is a disease caused by parasites of the *Leishmania* genus [223]. The high cost and low availability of antileishmanial drugs, as well as the developing resistance to these drugs, have made the current situation worrisome. However, this parasite is quite sensitive to AgNPs due to the generation of ROS. NPs possess combinatory action against *Leishmania tropica* under UV light [224]. Furthermore, it was observed that miltefosine-doped green AgNPs displayed an increased antileishmanial effect. Another study reported a similar promising activity (IC_50_ value 4.37) of green spherical AgNPs (3–8 nm) by employing *Sargentodoxa cuneata* [225]. The spherical AgNPs (116 nm) synthesized via *Moringa oleifera* extract showed a significant reduction in the average size of leishmaniasis cutaneous lesions in mice [226]. Despite these reports, further studies and trials are necessary to establish a concrete conclusion.

## 5. Conclusions and Future Perspectives

Scientists around the world usually seem skeptical when a given process employs a significant number of chemicals that may not be as safe as they seem to be, and this uncertainty has given rise to the now widely known concept of “green” processes. Owing to the drawbacks associated with synthetic approaches such as the employment of the reactive and toxic reducing and stabilizing agents that lead to adverse effects, a similar scenario has evolved in nanoparticle synthesis, especially silver nanoparticle fabrication methods. Responding to this challenge, the current review covered various ecofriendly AgNP synthesis methods including phytosynthesis, microbial-mediated synthesis, and enzyme-based synthesis, revealing the potential of various organisms and biomolecules to be employed as bionanofactories for green synthesis. The research has displayed that these bionanofactories are quite cost-effective and environmentally benign, while offering easy scale-up compared to conventional methods. For instance, the phytosynthesis methods revealed that the roots, shoots, bark, leaves, peel, flowers, and fruits of plants can be exploited as bionanofactories that consist of different phytoconstituents such as flavonoids, terpenoids, pectin, sugars, ascorbic acid, and carotenoids which aid in the synthesis by acting as reducing and capping agents, in addition to contributing to the therapeutic effect of the formulation. The fact that these plant extracts are easy to handle and readily accessible adds to the advantages of utilizing them in the current scenario. Additionally, microbes such as bacteria, fungi, yeast, algae, and actinomycetes can be employed for AgNP synthesis. Studies have demonstrated that the microbial synthesis method includes reactions such as trapping, bioreduction, and capping that can occur intracellularly or extracellularly. However, extracellular synthesis overcomes the limitations of the intracellular method by easing out the difficulties related to large-scale production, cumbersome purification, etc. In contrast to microbial synthesis, the phytosynthesis method seems advantageous as it does not require the complicated process of nurturing cell cultures, which makes it a more economic and industrially viable process.

Furthermore, the enzyme-based synthesis of AgNPs has revealed the potential of various enzymes such as α-NADPH-dependent nitrate reductase, as well as biomolecules such as glucose, galactose lactose, starch, heparin, and chitosan and vitamins such as vitamin B2 and vitamin C (ascorbic acid), to be employed as bionanofactories. Ionic liquid-mediated synthesis, the irradiation method, and microwave-assisted synthesis have introduced modernization to the green synthesis concept. In addition, the characterization of the synthesized AgNPs plays a crucial role in this process. This involves the determination of size, surface charge, distribution, surface morphology (shape), and aggregation as evaluated by employing a number of analytical techniques such as UV/visible spectroscopy, XRD, FTIR, SEM, AFM, TEM, DLS, and zeta potential analysis. Characterization studies, including in vitro and in vivo studies, have displayed the huge impact of the physicochemical properties of AgNPs on their therapeutic and biological applications.

Hence, we believe that the world is laden with an infinite number of biological species and compounds, some of which have been discovered while others are yet to be explored by humans. This contributes to the positive outlook of this emerging field and its immense potential to add value to the green methods currently available for the fabrication of AgNPs, which have already led to breakthroughs in a variety of fields ranging from therapeutics to diagnostics.

## Figures and Tables

**Table 1:** Use of bionanofactories in the synthesis of silver nanoparticles, along with a brief outline of their physicochemical and antimicrobial properties.

Category of Biofactories	Reducing Agent	UV/Vis	FTIR (cm^−1^)	Structural Analysis	In Vitro Activity Studies	Ref.
Green Algae	*Ulva compressa* *Cladophora glomerata*	403 nm (<6 h), 443 nm (after 24 h)	3278, 1634, 1539	AFM ▪ For AgNPs (*U. compressa*): Sa 1.01 nm; Sq 1.48 nm; Sz 9.09nm. ▪ For AgNPs (*C. glomerata*): Sa 0.471n m; Sq 0.848 nm; Sz 5.90 nm. XRD ▪ Crystalline ▪ Structure—face-centered cubic (FCC) Particle size ▪ AgNPs (*U. compressa*)—66.3 nm ▪ AgNPs (*C. glomerata*)—81.8	▪ Antimicrobial efficacy against species such as *K. pneumoniae*, *P. aeruginosa*, *E. coli*, *E. faecium*, and *S. aureus*	[36]
	*Spirogyra varians*	420–430 nm	3423, 2927, 1645, 1515, 1429	XRD ▪ Face-centered cubic (FCC) SEM ▪ Average size ~35 nm ▪ Shape—uniform and quasi-spherical	▪ Antibacterial *activity against *Staphylococcus aureus*, *Bacillus cereus*, Salmonella typhimurium, *Escherichia coli**, and *Listeria monocytogenes*	[37]
Brown Algae	*Spatoglossum asperum*	440 nm	1638, 1034, 3447, 1034.95, 1384.14	SEM ▪ Shape—spherical to oval ▪ Size—32 to 51 nm TEM ▪ Shape—mostly spherical ▪ Size—20 to 46 nm XRD ▪ Crystalline ▪ Structure—face-centered cubic (FCC) EDS ▪ Strong signal indicating presence of metallic silver	▪ Agar bioassay method showed reduction of bacterial colonies of *K. pneumoniae* with increasing concentration.	[38]
Red Algae	*Spyridia fusiformis*	450 nm	3907, 3779, 3410, 2927, 2853, 2593, 1644, 1416, 1170, 749	HR-TEM ▪ Shape—variable shapes such as spherical, triangle, pseudo-spherical, and some rounded rectangle shapes ▪ Most observed shape and size—spherical and 5 to 50 nm ▪ Average size of NPs—32.70 nm XRD ▪ Crystalline ▪ Structure—face-centered cubic (FCC)	▪ Antibacterial activity of AgNPs (concentration = 100 μg/mL) Maximum zone of inhibition: ▪ In *K. pneumoniae* = 26 mm ▪ In *S. aureus* = 24 mm	[39]
	*Gracilaria corticata*	424 nm; another peak at 220 nm maybe due to the presence of amide bond	2921, 1630, 1,455	EDS ▪ Optical absorption peak observed at 3 keV is typical for the absorption of metallic silver XRD ▪ Crystalline ▪ Structure—Face-centered cubic (FCC) TEM ▪ Size range = 10–35 nm	▪ Cytotoxic activity—on Hep2 cell lines, IC_50_ = 62.5 μg/mL	[40]
Blue-Green Algae	*Nostoc* sp.	419 nm	3443.96, 3385.61, 2923.83, 2853.32 1644.73	SEM ▪ Shape—spherical ▪ Average size 51–100 nm TEM ▪ Spherical and well-dispersed. TEM-SAED pattern ▪ Structure—face-centered cubic ▪ Nature—crystalline XRD ▪ Nature—crystalline	▪ No significant cytotoxicity against MCF-7 breast cancer cells at lower concentration; cytotoxicity increased with increasing concentration from 0 μL/mL to 50 μL/mL ▪ Good antibacterial and antifungal activity	[41]
	*Anabaena* sp.	420 nm	3383.72, 2930.60, 1651.13, 1076.58	TEM ▪ Size: 10–50 nm XRD ▪ Nature—crystalline ▪ Structure—face-centered cubic (FCC) ▪ Calculated particle size—20 nm	▪ Ag-CNP treatment inhibited growth of tumor and cancer cells and induced apoptosis	[42]
Actinomycetes	*Streptacidiphilus durhamensis*	-	3421, 1384.4, 1623, 1480	TEM ▪ Spherical shape EDS ▪ Indicated Ag as the major element with a ~3 keV signal ▪ Heterogeneous particle size distribution observed in the range of ~8–48 nm.	▪ Antimicrobial activity against the tested strains such as *S. aureus*, *B. subtilis*, *E. coli*, *P. aeruginosa*, *K. pneumoniae*, and *P. mirabilis* but not *S. infantis* ▪ Synergistic effects of bio(AgNPs) with various standard antibiotics	[43]
	*Streptomyces rochei*	410 nm	3420.14, 2932.23, 2362.37, 1639.20, 1430.92, 1115.62, 613.252	EDS ▪ Optical absorption peak for AgNPs observed at 3.5 keV SEM ▪ Size: 22 to 85 nm	▪ AgNPs exhibited synergistic effects with antibiotics such as ciprofloxacin, ampicillin, streptomycin, gentamicin, tetracycline and lincomycin ▪ AgNPs reduced the density of bacterial cells and acted as an antibiofouling agent	[44]
	*Streptomyces* sp.	425 nm	3695.61, 1585.49, 1398.39, 1151.50, 1068.56	XRD ▪ Nature—crystalline ▪ Structure—face-centered cubic (FCC) SEM ▪ Size range = 21–45 nm	▪ As compared to the cell-free supernatant, synthesized AgNPs showed high anticandidal activity	[45]
	*Nocardiopsis* sp. MBRC-1	420 nm	3440, 2923, 2853, 1655, 1460, 685	SEM ▪ Average particle size ~45 ± 0.05 nm FE-SEM and EDS ▪ The optical absorption peak was observed at 3 keV TEM ▪ Size range—30–90 nm ▪ Average particle size—45 ± 0.15 nm ▪ Shape—spherical	▪ Excellent antimicrobial activity by AgNPs observed against *Bacillus subtilis*, *Pseudomonas aeruginosa*, and *Candida albicans* ▪ Cytotoxicity studies against HeLa cancer cell lines, IC_50_ = 200 *μ*g/mL	[46]
Fungi	*Arthroderma fulvum*	420 nm	-	XRD ▪ Nature—crystalline ▪ Structure—face-centered cubic (FCC) TEM ▪ Shape—spherical ▪ Average diameter—15.5 ± 2.5 nm Particle size analysis ▪ Average diameter = 20.56 nm	▪ Potential antifungal activity against fungi such as *Candida* spp., *Aspergillus* spp., and *Fusarium* spp. observed ▪ Compared to antifungal drugs such as itraconazole and fluconazole, the biosynthesized AgNPs at concentrations near to 1 mg/mL showed a broader antifungal spectrum	[47]
	Mushroom *Pleurotus ostreatus*	400–470 nm	3318, 2944, 1612, 1411	HR-TEM and FE-SEM ▪ Shape—spherical in shape ▪ Average size range—10–40 nm Size distribution analysis ▪ Average size—28 nm EDS analysis ▪ 13% of Ag and rest presence of C & O recorded at 3 keV	▪ Antibacterial activity against *B. subtilis*, *B. cereus*, *S. aureus*, *E. coli*, and *P. aeruginosa* ▪ Bactericidal activity observed against *B. cereus*, *E. coli*, and *P. aeruginosa*	[48]
	*Raphanus sativus*	426 nm	3145, 1597, 1402, 1109, 1213, 995, 911, 699, 504	XRD ▪ Structure—face-centered cubic (FCC) ▪ Calculated mean size—~25 nm. TEM ▪ Shape—spherical ▪ Size range—10–30 nm SAED ▪ Nature—crystalline EDS ▪ Strong silver peaks at 3 keV AFM ▪ Monodispersed AgNPs, ▪ Average particle size 4 to 28 nm	▪ Antibacterial activity against human pathogenic bacteria such as *Bacillus subtilis*, *Staphylococcus aureus*, *Escherichia coli*, and *Serratia marcescens*	[49]
	Endophytic fungus *Curvularialunata*	422 nm	3430.86, 1573.16, 1483.37, 1402.84, 1260.95, 1123.70	SEM ▪ Shape—spherical ▪ Size (diameter) range—10 to 50 nm ▪ Average size—26 nm EDS ▪ Strong metal signal peak of Ag observed XRD ▪ Structure—face-centered cubic (FCC)	▪ Synthesized AgNPs, along with antibiotics, exhibited inhibitory activity against Gram-negative and Gram-positive bacterial pathogens such as *E. coli*, *Pseudomonas aeruginosa*, *Salmonella paratyphi*, *Bacillus subtillis*, *Staphylococcus aureus*, and *Bacillus cereus*	[50]
	*Penicillium* *polonicum*	430 nm	2920.23, 2850.79, 1747.51, 1508.33, 1473.62, 1338.60, 1361.74, 1240.16, 1035.77, 760.88	TEM ▪ Common shape—spherical ▪ Size range—10 to 15 nm ▪ Above size of 30 nm, hexagonal NPs HR-TEM ▪ Nature—crystalline SAED ▪ Nature—crystalline Particle size analysis ▪ Polydisperse AgNPs in the size range of 10–15 nm observed EDS ▪ Absorption peak recorded at 3 keV	▪ Killing kinetic assay depicted that complete killing of *A. baumanii* bacterial cells occurred within 6 h of exposure time to AgNPs	[51]
Bacteria	*Pseudomonas deceptionensis* DC5	428 nm	-	XRD ▪ Nature—crystalline FE-TEM ▪ Shape—spherical ▪ Size range—10 to 30 nm EDS ▪ A peak recorded at 3 keV Particle size analysis ▪ Average particles size—127 nm	▪ Activity efficiency observed in descending order against pathogens *V. parahaemolyticus*, *C. albicans*, *S. aureus*, *S. enterica*, and *B. anthracis* ▪ AgNPs at concentration of 5 μg/L found to inhibit biofilm formed by *S. aureus* and *P. aeruginosa*	[52]
	*Weissellaoryzae* DC6-	432 nm	-	FE-TEM ▪ Shape—spherical ▪ Size range—10 to 30 nm EDS ▪ Highest peak recorded at 3 keV XRD ▪ Nature—crystalline Particle size analysis ▪ Average particle size—150.2 nm ▪ Polydispersity index (PDI)—0.176	▪ Descending order of antimicrobial potential observed against *S. aureus*, *C. albicans*, *B. cereus*, *V. parahaemolyticus*, *E. coli*, and *B. anthracis* ▪ AgNPs at a concentration of about 5–6 μg found to inhibit the biofilm formed by *S. aureus* and *P. aeruginosa*	[53]
	*Bacillus thuringiensis*	413 nm	1644, 1549, 1520, 1114, 564, 550, 546, 523	FE-SEM ▪ Shape—spherical ▪ Average diameter range—10 to 30 nm TEM ▪ Size range 10 to 30 nm	▪ Purified AgNPs showed relatively stronger antibacterial activity against *E. coli* than the commercially available AgNPs	[54]
	Halotolerant *Bacillus endophyticus* SCU-L	420 nm	3400, 2969, 1650, 1560, 1453, 1401, 1227, 1083	XRD ▪ Structure—face-centered cubic TEM ▪ Shape—spherical ▪ Average size ~5.1 nm	▪ Antimicrobial activity observed against *C. albicans*, *E. coli*, *S. typhi*, and *S. aureus* ▪ AgNPs showed broad-spectrum antimicrobial activity against both Gram-positive and Gram-negative pathogens, as well as a fungus strain	[55]
	*Phenerochaete chrysosporium* (MTCC- 787)	430 nm	767, 1642, 2137,3400	TEM ▪ Shape—different shapes, such as spherical and oval ▪ Size range—34 to 90 nmAFM ▪ Agglomerated silver nanostructures	▪ Gram-negative clinical pathogens showed a higher susceptibility to AgNPs than Gram-positive pathogens	[56]
Plants (Roots)	*Delphinium denudatum*	416 nm	3354, 2952, 2063, 1651, 1419, 1383, 1354, 1171, 1093, 780, 672, 605	XRD ▪ Structure—face-centered cubic (FCC) ▪ Nature—crystalline FE SEM ▪ Spherical shape and size not more than 85 nm	▪ Antibacterial activity observed against *S. aureus*, *B. cereus* NCIM 2106, *E. coli*, and *P. aeruginosa* ATCC ▪ Susceptibility of dengue vector *aegypti* larvae to AgNPs increased when exposure time extended to 48 h; the LC_50_ values of AgNPs were 96 ppm (24 h) and 9.6 ppm (48 h) against second-instar larvae of *A. aegypti*	[56]
	*Alpinia katsumadai*	417 nm and during reaction 436 nm	3371–3377, 2980–2978, 1649–1653, 1389–1385, 1045–1049, 888-881	FE-TEM ▪ Shape—quasi-spherical ▪ Nature—well-dispersed and scattered EDS ▪ Strong absorption peak recorded at 3 keV XRD ▪ Structure—face-centered cubic ▪ Nature—crystalline	▪ Antibacterial activity observed against *S. aureus*, *E. coli*, and *P. aeruginosa*; thus, it was concluded that so-prepared AgNPs exhibited effective antioxidant, antibacterial, and anticancer activities	[57]
	*Aloe vera* leaves	420 nm	-	SEM ▪ Shape—spherical ▪ Size range—70.7 to 192.02 nm (size varied with temperature) XRD ▪ Structure—face-centered cubic (FCC)	▪ Minimal cytotoxicity to human PBMCs	[58]
	*Piper nigrum* leaf and stem	460 nm	3697, 3313, 3195, 2298, 1670, 1456, 1336, 1193, 1118, 811, 750, 651, 601	XRD ▪ Structure—face-centered cubic ▪ Nature—crystalline SEM ▪ Shape—spherical EDS ▪ Intense signal at 3 keV TEM ▪ Stem extracts of *P. nigrum* 9 to 30 nm ▪ Leaf extracts of *P. nigrum* - Small-sized AgNPs: 4 to 14 nm - Large-sized AgNPs: 20 to 50 nm	▪ Antibacterial activity—Stem and leaf synthesized AgNPs (at 50 *μ*L) showed activity against *Citrobacter freundii* and *Erwinia cacticida*	[59]
Plants (Fruit and Peel)	Banana peel	430nm	2353–2351, 1732–1755, 1640–1643, 1532–1537, 1445–1454	SEM–EDS ▪ A distinct signal and high atomic percent values for silver were obtained XRD ▪ Structure—face-centered cubic (FCC) ▪ Nature—crystalline	▪ AgNPs exhibited potent antifungal activity against the tested pathogenic strains of *C. albicans* and *C. lipolytica* ▪ The antibacterial activity of AgNPs was observed against *E. coli*, E. aerogenes, Klebsiella sp., and Shigella sp.	[60]
	*Tribulus terrestris* dried fruit	435 nm	-	XRD ▪ Nature—crystalline AFM ▪ Shape—spherical ▪ Particle size ~24.631 nm TEM ▪ Shape—spherical shape ▪ Average size—22 nm	▪ Antimicrobial activity against *S. pyogens*, *S. aureus*, *B. subtilis*, *P. aeruginosa*, and *E. coli*.	[61]
	Lemon	400–430 nm	-	AFM ▪ Particle dimensions—height 12 nm, width 100 nm ▪ SEM—NPs consisted of agglomerates of small grains with diameter of approximately 75 nm	▪ Disc diffusion method showed that NPs reduced the growth of both *E. coli* and *Bacillus subtilis*	[62]
Other biosynthesizing Agents	Gum kondagogu (*Cochlospermum gossypium*)	416 nm	3443, 2916, 2850, 1727, 1630, 1597, 1384, 1351, 1254, 1148, 1043	TEM ▪ Shape—anisotropic nanostructures such as nanotriangles, a few nanorods, hexagonal and polygonal nanoprisms, and abundant unevenly shaped nanoparticles were observed ▪ Nature—polydisperse ▪ For 30 min of reaction time, size of 55.0 nm; for 60 min of reaction time, size of 18.9 nm SAED ▪ Nature—crystalline ▪ Structure—face-centered cubic (FCC) TEM ▪ Shape—spherical ▪ Average particle size: for 30 min of reaction time, 11.2 nm; for 60 min of reaction time, 4.5nm XRD ▪ Nature—crystalline ▪ Structure—face-centered cubic	▪ Antibacterial activity was observed against *S. aureus* ATCC 25923, *E. coli* ATCC 25922, *E. coli* ATCC 35218, and *P. aeruginosa* ATCC 27853	[63]
	Dextran T40	423 nm	-	AFM ▪ Particle size range—10 to 60 nm TEM ▪ Shape—spherical ▪ Size ~5–10 nm SAED ▪ Nature—crystalline. EDS ▪ Optical absorption peak at 3 keV XRD ▪ Structure—face-centered cubic (FCC)	▪ Antimicrobial activity observed against *B. subtilis*, *B. cereus*, *E. coli*, *S. aureus*, and *P. aeruginosa*.	[64]
	Casein (milk protein)	400 to 500 nm	1644, 1514	SEM and TEM ▪ Shape—spherical agglomerates formed upon carefully decreasing the pH to 3.32 ▪ Size—average diameter of about 60 to 80 nm	▪ AgNPs at a dose of 0.025 µg/mL, i.e., below LD_50_ value, was observed to be fairly distributed in cytoplasm of living cells imaged by CLSM	[65]

Sa—average roughness, Sq—root-mean-square roughness, Sz—ten-point height.
